# Study of *Holtermanniella wattica*, *Leucosporidium creatinivorum*, *Naganishia adeliensis*, *Solicoccozyma aeria,* and *Solicoccozyma terricola* for their lipogenic aptitude from different carbon sources

**DOI:** 10.1186/s13068-016-0672-1

**Published:** 2016-11-28

**Authors:** Sara Filippucci, Giorgia Tasselli, Alessandro Scardua, Simone Di Mauro, Maria Rita Cramarossa, Davide Perini, Benedetta Turchetti, Andrea Onofri, Luca Forti, Pietro Buzzini

**Affiliations:** 1 Department of Agricultural, Food and Environmental Sciences & Industrial Yeasts Collection DBVPG, University of Perugia, Borgo XX Giugno 74, 06121 Perugia, Italy; 2 Laboratories of Biotechnology, Novamont S.p.A, via Fauser 8, Novara, 28100 Italy; 3 Department of Life Sciences, University of Modena and Reggio Emilia, via Campi 287, Modena, 41125 Italy

**Keywords:** Oleaginous yeasts, Lipid yield, Fatty acid profile, Large-scale screening, *Leucosporidium creatinivorum*

## Abstract

**Background:**

The ability of some microorganisms to accumulate lipids is well known; however, only recently the number of studies on microbial lipid biosynthesis for obtaining oleochemical products, namely biofuels and some building blocks for chemistry, is rapidly and spectacularly increased. Since 1990s, some oleaginous yeasts were studied for their ability to accumulate lipids up to 60–70% of their dry weight. Due to the vast array of engineering techniques currently available, the recombinant DNA technology was the main approach followed so far for obtaining lipid-overproducing yeasts, mainly belonging to the *Yarrowia lipolytica*. However, an alternative approach can be offered by worldwide diversity as source of novel oleaginous yeasts. Lipogenic aptitude of a number of yeast strains has been reviewed, but many of these studies utilized a limited number of species and/or different culture conditions that make impossible the comparison of different results. Accordingly, the lipogenic aptitude inside the yeast world is still far from being fully explored, and finding new oleaginous yeast species can acquire a strategic importance.

**Results:**

*Holtermanniella wattica*, *Leucosporidium creatinivorum*, *Naganishia adeliensis*, *Solicoccozyma aeria,* and *Solicoccozyma terricola* strains were selected as a result of a large-scale screening on 706 yeasts (both Ascomycota and Basidiomycota). Lipid yields and fatty acid profiles of selected strains were evaluated at 20 and 25 °C on glucose, and on glycerol, xylose, galactose, sucrose, maltose, and cellobiose. A variable fatty acid profile was observed in dependence of both temperature and different carbon sources. On the whole, *L. creatinivorum* exhibited the highest performances: total lipid yield (Y_L_) >7 g/l on glucose and glycerol, % of intracellular lipids on cell biomass (Y_L_/DW) >70% at 20 °C on glucose, lipid coefficient (Y_L_/Glu) around 20% on glucose, and daily productivity (Y_L_/d) on glucose and sucrose >1.6 g/(l*d).

**Conclusions:**

This study provides some meaningful information about the lipogenic ability of some yeast species. Variable lipid yields and fatty acid profiles were observed in dependence of both temperature and different carbon sources. *L. creatinivorum* exhibited the highest lipogenic performances.

**Electronic supplementary material:**

The online version of this article (doi:10.1186/s13068-016-0672-1) contains supplementary material, which is available to authorized users.

## Background

The ability of some microorganisms to accumulate high intracellular amounts of lipids has been known for decades; however, only recently the number of studies on microbial lipid biosynthesis for obtaining oleochemical products, namely biofuels and some building blocks for lubricants, adhesives, solvents, biosurfactants, cosmetics, and degradable polymers, has rapidly and spectacularly increased, becoming a growing part of the so-called “white biotechnology” [1–4]. In particular, the production of vegetable and microbial lipids is largely connected to the advent of biodiesel, which is considered one of the most promising biofuels [1–3]. Biodiesel is derived by homogeneously or heterogeneously catalyzed trans-esterifications of triacylglycerols (TAGs). The increasingly global request of biodiesel from vegetable oils occurred from early 2000s, and it has caused the dramatic increase of market prices for a number of foodstuffs. Moreover, vegetable lipids are considered one of the most important renewable feedstocks of the chemical industry since they could be processed by means of chemical routes and/or biotechnology approaches to produce high-value chemical compounds and non-fuel oil-derived products [5–7].

Accordingly, finding new biological sources of lipids acquires a strategic importance to reduce (or even to avoid) any competition with food resources [8, 9]. In this framework, microbial lipids are among the most promising feedstock sources for oil production because their composition is quite similar to that found in most vegetable oils [10]. Besides, taking into consideration the possibility to store big amounts of feedstock surpluses, the production of lipids via microorganisms has a lot of advantages, namely quite a simple process due to their short life cycle, no seasonal and climatic influences, and a greater ease to scale-up the process [11–17].

Yeasts are a well-known group of eukaryotic organisms belonging to the Kingdom of fungi that are currently exploited both for traditional and innovative uses [18, 19]. Since 1990s, some yeast species, in particular those belonging to the genera *Yarrowia*, *Lipomyces*, *Cryptococcus*, *Rhodotorula*, *Rhodosporidium,* and *Trichosporon* (otherwise labelled as “oleaginous yeasts”), were studied for their ability to accumulate lipids up to 60–70% of their dry weight. However, those species represent only a tiny fraction of the total yeast diversity; additionally, it was found that only 5% of the yeasts so far studied were able to accumulate lipids for more than 25% of their dry weight [4, 10–12, 14, 20, 21].

Lipids produced by oleaginous yeasts are TAGs rich in monounsaturated (MUFAs) and polyunsaturated (PUFAs) fatty acids [22]. TAGs are accumulated in the yeast cytoplasm into hydrophobic lipid particles (droplets), which can be used by cell metabolism for membrane biosynthesis and as an energy reserve [23–26]. Lipid accumulation is usually obtained from different carbon sources, using substrates characterized by high carbon and limited nitrogen availability; after the complete depletion of nitrogen, the growth rate slows down and in the case of oleaginous yeasts the residual carbon source is channeled toward lipid synthesis, leading to intracellular lipid accumulation [11, 12, 27].

Due to the vast array of engineering techniques currently available, the recombinant DNA technology was the main approach followed so far for obtaining lipid-overproducing yeasts; in this contest, the species *Yarrowia lipolytica* was the most studied oleaginous yeast [1, 19, 28–34]. However, an alternative approach can be offered by yeast worldwide diversity as source of novel oleaginous yeasts [4, 11, 12]. Some reviews highlight that a number of wild yeasts, belonging to both Ascomycota and Basidiomycota taxa, exhibit important metabolic activities that can play an important role in biotechnology, offering an alternative to conventional yeasts [18, 19].

Lipid content and/or fatty acid composition of a number of yeast strains have been reviewed [10–12, 35]. However, many of these studies used a limited number of species. Additionally, differences in culture conditions make the comparison of results from different studies impossible, as lipid yields depend highly on culture conditions, namely carbon and nitrogen sources, C/N molar ratio, temperature, and oxygenation [4, 10–12, 14, 35, 36]. Accordingly, the lipogenic aptitude inside the yeast world is still far from being fully explored.

In this framework, the screening of a large set of yeasts isolated from worldwide sources was used to select strains capable of producing high amounts of lipids using different carbon sources.

## Methods

### Chemicals

All chemicals used in the study were from Carlo Erba (Milano, Italy), while media were from Oxoid (Roskilde, Denmark), unless otherwise stated.

### Microorganisms

Seven hundred and six yeast strains belonging to 45 genera and 86 species (284 ascomycetous strains belonging to 25 genera and 45 species and 422 basidiomycetous strains belonging to 20 genera and 41 species) isolated from Europe, North and South America, Africa, Asia, and Antarctica were used. About 75% of them were isolated from natural environments, 16% from foodstuffs, 1% from human-associated habitats, while the isolation source was unknown for 8% of the strains. Based on their optimum, minimum, and maximum growth temperatures [37], 67% were mesophiles (over two-thirds of which were ascomycetes and the remaining basidiomycetes), 30% were psychrotolerant (all basidiomycetes) and 3% were psychrophiles (all basidiomycetes). Two bench marker strains, i.e., *Saccharomyces cerevisiae* DBVPG 6173 (corresponding to CBS 1171, which was considered as non-lipogenic strain) and *Y. lipolytica* DBVPG 6053 (CBS 6124, as lipogenic strain), were also comparatively used to further strengthen the meaning of the data collected in this study. Both strains were selected because they are the type strains of their own species. All strains are preserved in the Industrial Yeast Collection DBVPG of the Department of Agricultural, Food and Environmental Sciences, University of Perugia, Italy. Salient information on yeasts used in this study is reported in the Supplementary materials (Additional file 1: Table S1) and on the DBVPG website (http://www.dbvpg.unipg.it). Working cultures were sub-cultured on YPD agar: 20 g/l glucose, 10 g/l yeast extract, 10 g/l peptone, 20 g/l agar, pH 6.0.

### Primary screening for lipid production

A flow chart figure summarizing the workflow of the study is reported in Supplementary materials (Additional file 2: Figure S1). A loopful of 48-h yeast cultures grown on YPD agar (OD_600_ adjusted to 0.1, average cell concentration = 10^5^ cells/ml) were used to inoculate glass tubes containing 5 ml of GMY broth: 40 g/l glucose, 8 g/l KH_2_PO_4_, 0.5 g/l MgSO_4_.7H_2_O (both salts from Sigma-Aldrich, Dorset, UK), 3 g/l yeast extract, pH 5.5, C/N ratio = 40 [38]. The tubes were incubated at 15, 20, and 25 °C for 15, 9, and 7 days, respectively (according to the optimal growth temperature of each species reported in Additional file 1: Table S1). Cell growth was quantified by OD_600_.

During the primary screening, the intracellular lipid accumulation was evaluated using the method described in Sitepu et al. [4, 39] using the fluorescent dye Nile Red (9-diethylamino-5H-benzo-α-phenoxazine-5-one) (Sigma-Aldrich). Nile Red stock solution (0.1 mg/ml in acetone) was prepared immediately before use, and an aliquot (0.04 ml) of stock solution was added to 1 ml of each yeast culture collected in an Eppendorf tube. After five minutes, a drop of cell suspension was observed by a DMLB light microscope (Leica Microsystems, Buffalo Grove, IL, USA) equipped with a 301-185.104-00 lamp, 40× and 63× objectives, and L5, I3, A, N2.1 fluorescence filters (excitation and emission wavelengths of 530 and 580 nm, respectively). Cells of all yeast strains used in this study were photographed before and during fluorescence emission with a Wild MP 552 camera (Leica Microsystems) (Additional file 3: Figure S2). The freeware ImageJ (http://imagej.nih.gov/) was used to achieve an estimation both of the intensity of fluorescence emitted by each strain and of the % of cell area occupied by lipid particles by capturing and processing cell images.

Accordingly, the amount of intracellular lipids was estimated by correlating the intensity of fluorescence emitted by each strain and the % of cell area occupied by lipid particles using the following formula:

$${\text{EILY}} = {\text{IEF}} \times {\text{TLPA}}/{\text{CA}},$$

where EILY is estimated intracellular lipid yield, IEF is intensity of emitted fluorescence, TLPA is total lipid particle area, and CA is cell area.

The amount of biomass produced after cultivation was determined gravimetrically as cell dry weight (DW) by following the protocol suggested by Kitcha and Cheirsilp [40].

### Secondary screening for lipid production

A loopful of 48-h yeast cultures grown on YPD agar (OD_600_ adjusted to 0.1) of 71 yeast strains belonging to the basidiomycetous species (selected after the primary screening) were inoculated in 50-ml shaken flasks containing 10 ml of YPD broth and incubated in an orbital shaker (160 rpm) at 20 and 25 °C for 3 and 2 days, respectively (according to the optimal growth temperature of each species reported in Additional file 1: Table S1). After incubation, 1 ml of the yeast cultures (OD_600_ adjusted to 0.1) were inoculated in 250-ml shaken flasks containing 50 ml of GMY broth and incubated in an orbital shaker (160 rpm) at 20 and 25 °C until the complete depletion of glucose. Yeast growth during batch cultivation was daily monitored by measuring OD_600_. The amount of biomass produced after batch cultivations was determined gravimetrically as DW.

Glucose depletion during batch cultivation was monitored with the commercial K-GLUC 07/11 kit (Megazyme, Chicago, IL, USA) following the supplier’s protocol.

The extraction of intracellular lipids was done using the protocol of Li-Xia et al. [25] with a few modifications. Briefly, 20 ml of each culture was centrifuged at 5000×*g* for 10 min. After centrifugation and subsequent washing, the cells were thus treated with 10 ml of 4 M HCl (Chem-Lab Analytical, Zedelgem, Belgium) and incubated at 60 °C for 2 h in a water bath. The acid-hydrolyzed biomass was mixed with 15 ml of a chloroform/methanol 2:1 (v/v %) mixture and incubated at room temperature for 2 h in an orbital shaker (160 rpm). Samples were then centrifuged at 4000×*g* for 10 min to favor the separation of phases. The phase containing the lipids was thus gently recovered and put into 25-ml glass vials, which were immediately sealed with a rubber septum and lead to dryness in the dark under a gas nitrogen flow. The total amount of lipids produced by strains after batch cultivations was weighted using an Extend Analytical Balance (Sartorius, Goettingen, Germany).

### Evaluation of lipid production at different temperatures

A loopful of 48-h yeast cultures grown on YPD agar (OD_600_ adjusted to 0.1) of the strains selected after the secondary screening, and of *S. cerevisiae*- and *Y. lipolytica*-type strains, were inoculated in 50-ml shaken flasks containing 10 ml of YPD broth and incubated in an orbital shaker (160 rpm) at 20 and 25 °C for 3 and 2 days, respectively. After incubation, 1 ml of yeast cultures (OD_600_ adjusted to 0.1) was inoculated in 250-ml shaken flasks containing 50 ml of GMY broth and incubated in an orbital shaker (160 rpm) at both 20 and 25 °C until the complete depletion of glucose. All batches were carried out in triplicate.

High-resolution images of Nile Red staining of *Leucosporidium creatinivorum* DBVPG 4794 (incubated at 20 °C), *Naganishia adeliensis* DBVPG 5195, and *Solicoccozyma terricola* DBVPG 5870 (both at 25 °C) were realized with an UV epifluorescence microscope Olympus BX53 (Olympus Co., Centre Valley, PA, USA) equipped with excitation filter BP470-500 and barrier filter BA515-560. Cells of the above three yeast strains were photographed before and during fluorescence emission with a XC50 camera (Olympus Co.) (Additional file 4: Figure S3).

During batch cultivation, the glucose depletion was monitored with the commercial K-GLUC 07/11 kit (Megazyme), while yeast growth was daily monitored by measuring OD_600_. The final amount of biomass was determined gravimetrically as DW.

The extraction of intracellular lipids was done as above reported, and vials containing lipids were thus frozen and stored at −20 °C until GC–MS analysis.

### Evaluation of lipid production from C3, C5, C6, and C12 carbon sources

Glycerol (C3), xylose (C5), galactose (C6), sucrose, maltose, and cellobiose (C12) were used instead of glucose (at the same molar concentration) to obtain modified GMY broth. A loopful of 48-h yeast cultures grown on YPD agar (OD_600_ adjusted to 0.1) were inoculated in 50-ml shaken flasks containing 10 ml of YPD broth and incubated in an orbital shaker (160 rpm) at 20 and 25 °C for 3 and 2 days, respectively. After incubation, 1 ml of yeast cultures (OD_600_ adjusted to 0.1) was inoculated in 250-ml shaken flasks containing 50 ml of modified GMY broth. Strains were incubated in an orbital shaker (160 rpm) at their optimal growth temperature for complete depletion of the carbon source. All batches were carried out in triplicate.

Carbon source (glycerol, xylose, galactose, maltose, and sucrose) depletion was monitored with the commercial kits K-GCROL 07/12 (glycerol), K-XYLOSE 08/14 (xylose), K-ARGA 02/15 (galactose), and K-MASUG 02/15 (maltose and sucrose) (Megazyme) following the supplier’s protocol. A different method was used to monitor cellobiose depletion: the commercial enzyme ß-glucosidase supplied with E-BGOSAG kit (Megazyme) was diluted 1:10 in 50 mM sodium maleate buffer at pH 6.5 in the presence of 0.5 mg/ml of Bovine Serum Albumin (Sigma-Aldrich). After incubation at 40 °C for 15 min, the glucose released by cellobiose hydrolysis was quantified with the K-GLUC 07/11 kit.

Yeast growth, the amount of biomass produced after batch cultivations, and the amount of intracellular lipids were determined as reported above.

Also in this case, vials containing lipids were thus frozen and stored at −20 °C until GC–MS analysis.

### Evaluation of the main parameters for lipid production in selected strains

Depending on the carbon sources used, the following parameters were determined:

- the overall (volumetric, g/l) lipid production (total lipid yield = Y_L_);
- the % of total intracellular lipids on cell biomass (Y_L_/DW);
- the lipid coefficient (Y_L_/carbon source) = the total lipid yield for the specific carbon source used (e.g., Y_L_/Glu = the lipid coefficient when glucose was used); and
- the daily productivity [g/(l*d)] = the lipid yield produced per day (Y_L_/d).

### Evaluation of fatty acid profile of lipids

Fatty acid composition was analyzed qualitatively and quantitatively by gas chromatographic analysis after a one-step procedure of direct esterification of lipids plus extraction.

A portion of lipid sample (3–5 mg) was dissolved in 2 ml of hexane containing 0.5 mg/ml of methyl benzoate as internal standard and poured into a Schlenk tube with Teflon cap. 2 ml 15% H_2_SO_4_ in methanol was added for the trans-esterification step. Each sample was heated up to 100 °C for 1 h with continuous shaking. After cooling on ice, the samples were centrifuged (3500×*g*), and 1 μl of the upper phase, containing the fatty acid methyl esters extract, was analyzed via gas chromatography/mass spectrometry (Agilent Technologies, 5890 Series II–5972 Mass Selective Detector) equipped with a HP-5 column (25 m × 0.2 mm, 0.5 μm film thickness) coated with (5%)-diphenyl-(95%)-dimethylpolysiloxane copolymer. Fatty acid methyl esters were identified by comparing their respective mass fragmentation patterns (EI, 70 eV) with the database library NIST05 (MS Library Software, Varian, USA). Temperature program was as follows: 40 °C, hold for 1 min; 8 °C/min to 240 °C, hold for 10 min; injector 270 °C; detector 280 °C.

Based on the fatty acid profile of the different strains, the Unsaturation Index (UI) was calculated by the Watson’s formula reported by Vishniac [41]:

$${\text{UI}} = \left[ {\% {\text{ monoenes}} + 2\left( {\% {\text{ dienes}}} \right) + 3\left( {\% {\text{ trienes}}} \right)} \right]/ 100.$$

### Statistical analysis

Batch fermentations were carried out in triplicate, and, wherever necessary, statistical testing was carried out using non-parametric Kruskal–Wallis tests or ANOVA. Principal Component Analysis (PCA) was carried out on the fatty acid profiles using the R environment for statistical computing [42]. Data were standardized prior to analysis, and results were displayed on a correlation biplot [43].

## Results and discussion

### Screening and selection of lipid-overproducing yeasts

A considerable inter- and intraspecific variability of the capacity to accumulate high levels of intracellular TAGs was observed, in agreement with previous studies [4, 11, 12]. The 2D scatter plot reporting the correlation between EILY (calculated as reported in sub-paragraph “Primary screening for lipid production”) and DW exhibited a quite dispersed distribution of the 706 tested strains (Additional file 5: Figure S4). This is apparently in agreement with some studies underlining that the lipogenic aptitude of yeasts is not clade-specific [4, 11, 12, 35, 36, 39]. However, both taxon- and temperature-related trends were found in the 706 strains used in this study. In fact, the EILY and DW values for the 706 strains categorized on the basis of their taxonomic position (Ascomycota *vs* Basidiomycota) showed that the two centroids (Xa and Xb = centroids of ascomycetes and basidiomycetes, respectively) laid on different positions, implying a superior performance of Basidiomycota (Additional file 5: Figure S4A). Kruskal–Wallis test confirmed that the median EILY and DW values for the two taxonomic groups were significantly different (*P* < 10^−16^, for both variables). Besides, lipid and biomass accumulation of all 422 basidiomycetous strains were also categorized on the basis of their optimal growth temperature (psychrophiles vs psychrotolerant vs mesophiles). Interestingly, although a quite dispersed distribution was observed also in this case, the position of the three centroids (Xp, Xpt, and Xm = centroid of psychrophiles, psychrotolerant, and mesophiles, respectively) suggested that psychrotolerant and, to a lesser extent, mesophiles exhibited a superior lipogenic aptitude (Additional file 5: Figure S4B). Also in this case, Kruskal–Wallis tests confirmed that the median EILY and DW values for the three optimal temperatures were significantly different (*P* < 0.001).

As a result of this primary screening, 71 lipid-overproducing strains (40 psychrotolerant and 31 mesophiles) belonging to Basidiomycota were selected for further experiments. The selected strains belonged to different species, some of which have been recently taxonomically revised by multigene sequence analyses and assigned to different genera of Pucciniomycotina and Tremellomycetes clades [44, 45]: *Buckleyzyma aurantiaca* (former *Rhodotorula aurantiaca*)*, Holtermanniella wattica, Leucosporidium creatinivorum* (former *Leucosporidiella creatinivora*), *Naganishia adeliensis* (former *Cryptococcus adeliensis*)*, Naganishia albida* (former *Cryptococcus albidus*)*, Rhodotorula mucilaginosa, Solicoccozyma aeria* (former *Cryptococcus aerius*)*, Solicoccozyma terreus* (former *Cryptococcus terreus*)*, Solicoccozyma terricola* (former *Cryptococcus terricola*)*, Tausonia pullulans* (former *Guehomyces pullulans*), and *Vanrija humicola* (former *Cryptococcus humicola*). All 71 selected strains were isolated from environmental sources, confirming the potential of this portion of yeast biodiversity as important (and understudied) source of biochemicals, including lipids. This set of new oleaginous yeasts included strains isolated and deposited in the Industrial Yeast Collection DBVPG in the last years, as well as strains maintained for over seven decades, emphasizing the importance of long-term preservation of biodiversity in biological culture collections for future research, as recently highlighted [46].

**Fig. 1:**
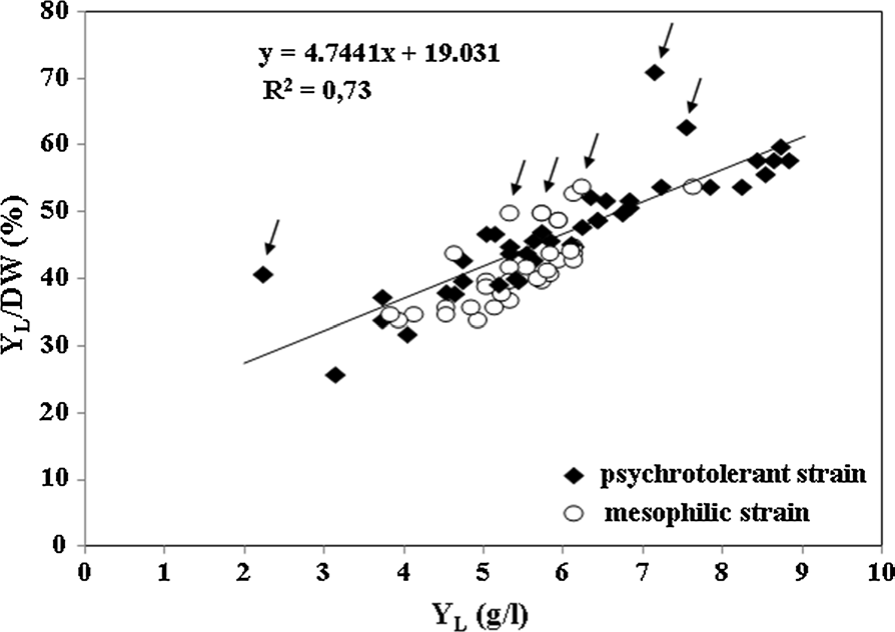
2D scatterplot of basidiomycetous oleaginous yeasts as a function of their lipogenic aptitude. *Y*_*L*_ total lipid yield (g/l), *Y*_*L*_ */DW* % of intracellular lipids on cell dry weight. Media: GMY; Temperature: psychrotolerant strains: 20 °C; mesophilic strains: 25 °C; Incubation time: 9 and 7 days for psychrotolerant and mesophilic strains, respectively

A linear relationship (${\text{y}} = 4. 7 4 4 1 {\text{x}} + 1 9.0 3 1$; *R* ^2^ = 0.73) between Y_L_ and Y_L_/DW was observed in the 71 selected yeasts (Fig. 1). Overall, the psychrotolerant strains showed a wider distribution (from lower to higher values of both Y_L_ and Y_L_/DW), while mesophilic ones exhibited a more concentrated distribution around mean values of both Y_L_ and Y_L_/DW. However, taking into consideration only the strains showing values of Y_L_ and Y_L_/DW superior to 6 g/l and 50%, respectively, it is possible to observe that psychrotolerant strains exhibited a superior performance than mesophiles (Fig. 1).

The relationship between Y_L_ and Y_L_/DW herein reported deserves further discussion because Y_L_ and the Y_L_/DW are currently used as interchangeable criteria for selecting lipid-overproducing yeasts [11, 12]. However, we found that although the linear equation summarizes quite well the relationship between Y_L_ and Y_L_/DW, some strains did not follow this trend, because they exhibited higher Y_L_/DW, but lower Y_L_ (Fig. 1, see strains highlighted by arrows). This evidence suggests that an increase of Y_L_/DW is not always related to a proportional increase of Y_L_. This result raises the issue of what criteria should be taken into consideration for screening lipogenic ability of yeasts. Based on the above results, we could conclude that Y_L_ (expressed as g/l) is apparently more correlated with the general yeast lipogenic aptitude than Y_L_/DW. Accordingly, our results highlight that Y_L_/DW cannot be used as the main (or even exclusive) criterion for selecting lipid-overproducing yeasts, and that other parameters correlated with Y_L_, namely DW, must not be ignored, as suggested by a previous study [4].

Interestingly, the prevalence of psychrotolerant strains at higher values of both Y_L_ and Y_L_/DW apparently confirmed a few studies reporting that the lipogenic ability of yeasts is also correlated with their growth optimal temperature [15, 47].

**Fig. 2:**
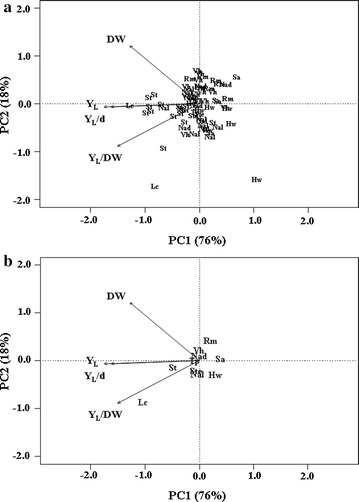
PCA of basidiomycetous oleaginous yeasts as a function of their lipogenic aptitude. *Y*_*L*_ total lipid yield (g/l), *DW* cell dry weight (g), *Y*_*L*_ */DW* % of intracellular lipids on DW; *Y*_*L*_ */d* total lipid yield daily productivity [g/(l*d)]; **a** PCA of 71 basidiomycetous strains; **b** PCA of centroids of the 71 basidiomycetous strains; Ba, *Buckleyzyma aurantiaca*; Hw, *Holtermanniella wattica*; Lc, *Leucosporidium creatinivorum*; Nad, *Naganishia adeliensis*; Na, *Naganishia albida*; Rm, *Rhodotorula mucilaginosa*; Sa, *Solicoccozyma aeria*; Ste, *Solicoccozyma terreus*; St, *Solicoccozyma terricola*; Tp, *Tausonia pullulans*; Vh, *Vanrija humicola*. Media: GMY; Temperature: psychrotolerant strains: 20 °C; mesophilic strains: 25 °C; Incubation time: 9 and 7 days for psychrotolerant and mesophilic strains, respectively

Principal component analysis (PCA) was used to check the correlations among the 71 strains and their Y_L_, DW, Y_L_/DW, and Y_L_/d (Fig. 2). PC1 and PC2 accounted for about 76 and 18% of variance, respectively. Overall, PCA revealed a huge intraspecific variability of lipid production (Fig. 2a). However, taking into consideration only the centroids of each species, the position of Y_L_, Y_L_/DW, and Y_L_/d suggests the superior lipogenic aptitude of the species *L. creatinivorum* (Fig. 2b).

### Lipid production at different temperatures

**Fig. 3:**
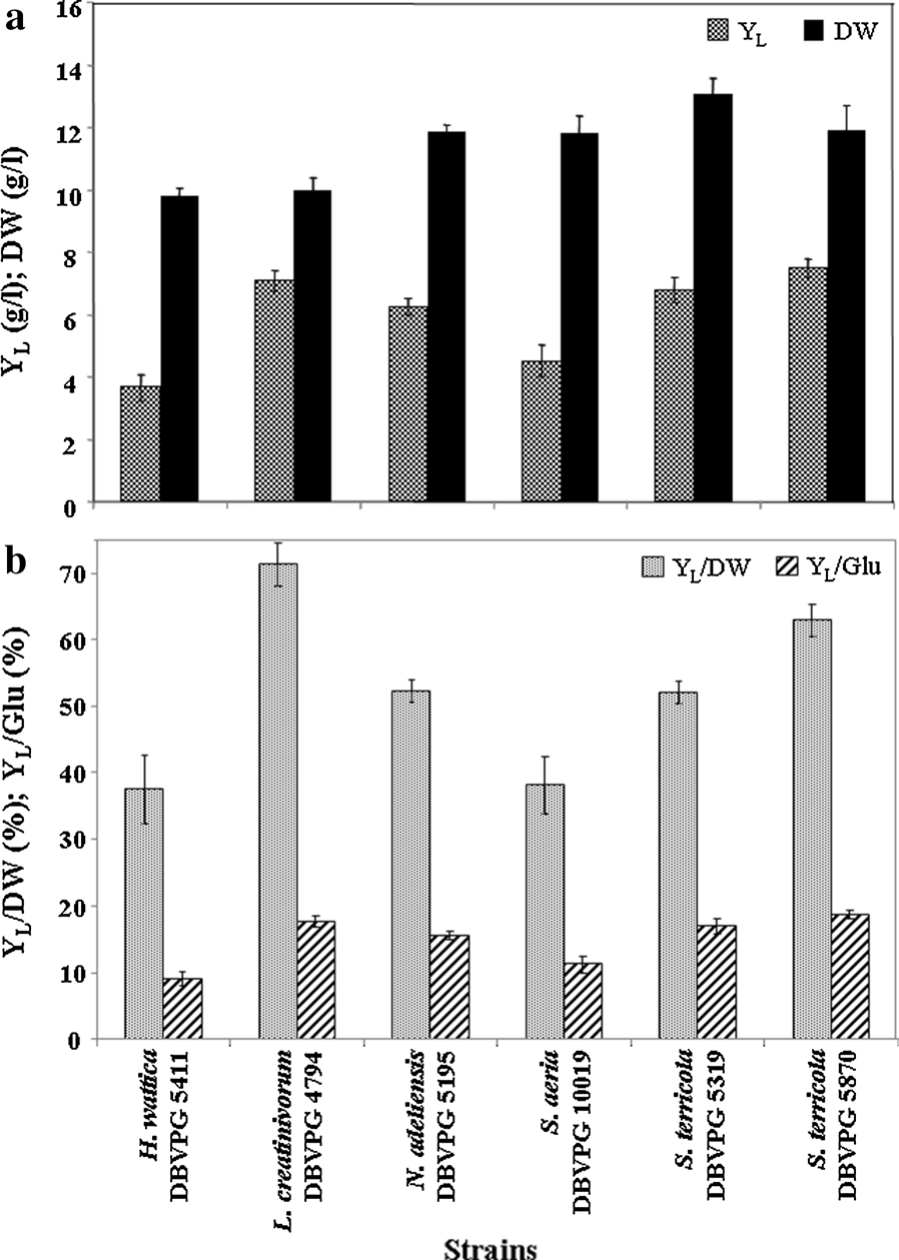
Lipid and biomass yields of selected yeast strains grown at 20 °C. Strains: *Holtermanniella wattica* DBVPG 5411, *Leucosporidium creatinivorum* DBVPG 4794, *Naganishia adeliensis* DBVPG 5195, *Solicoccozyma aeria* DBVPG 10019, *Solicoccozyma terricola* DBVPG 5319 and DBVPG 5870 **a** *Y*_*L*_ total lipid yield (g/l), *DW* cell dry weight (g), **b** *Y*_*L*_ */DW* % of intracellular lipids on DW, *Y*_*L*_ */Glu* % of lipid produced in 100 g of glucose. Media: GMY; temperature: 20 °C; incubation time: until the total glucose depletion (DBVPG 4794: 4 days; DBVPG 5195 and 5870: 6; DBVPG 5319: 8; DBVPG 5411; and 10019: 9)

**Fig. 4:**
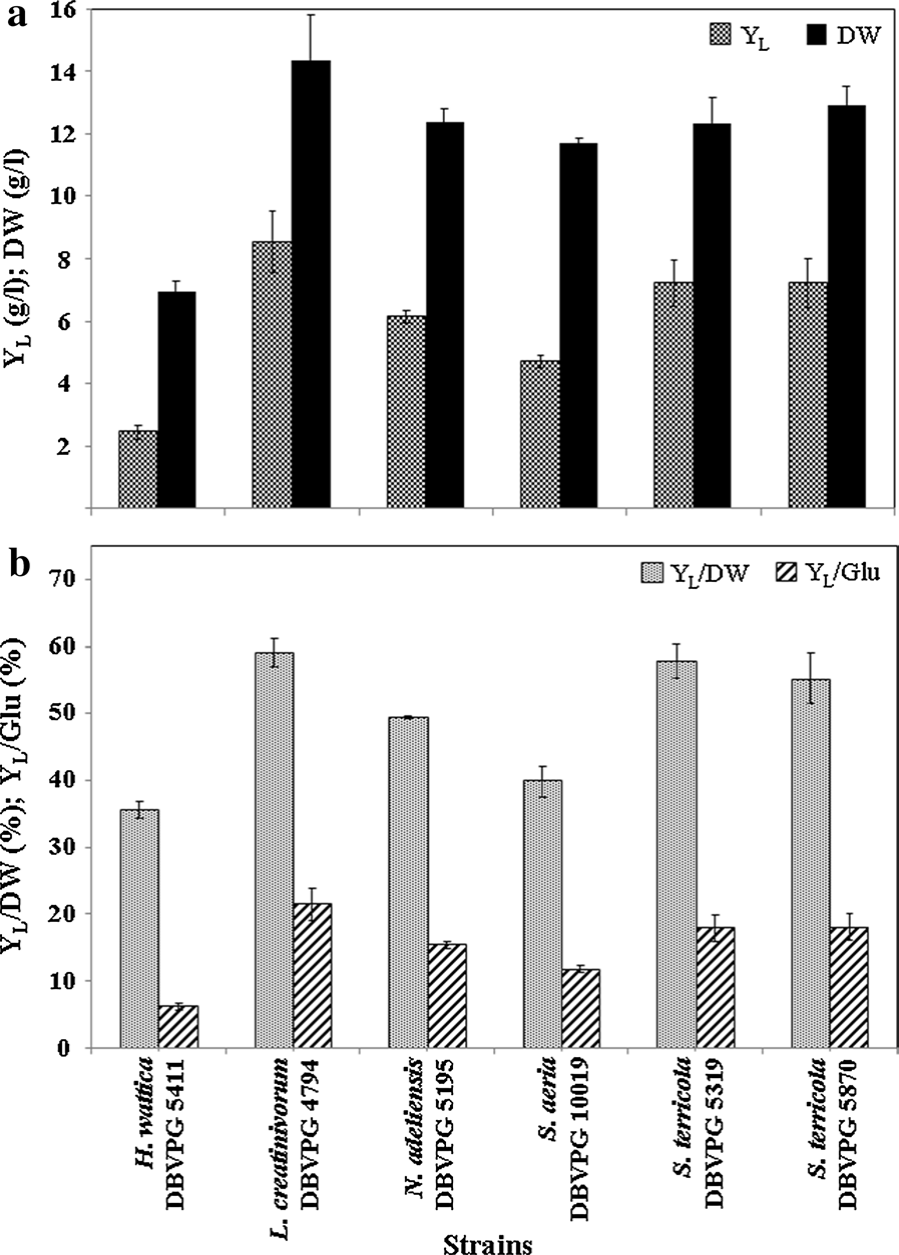
Lipid and biomass yields of selected yeast strains grown at 25 °C. Strains: *Holtermanniella wattica* DBVPG 5411, *Leucosporidium creatinivorum* DBVPG 4794, *Naganishia adeliensis* DBVPG 5195, *Solicoccozyma aeria* DBVPG 10019, *Solicoccozyma terricola* DBVPG 5319 and DBVPG 5870. **a** *Y*_*L*_ total lipid yield (g/l), *DW* cell dry weight (g), **b** *Y*_*L*_ */DW* % of intracellular lipids on DW; *Y*_*L*_ */Glu* % of lipid produced in 100 g of glucose. Media: GMY; temperature: 25 °C; incubation time: until the total glucose depletion (DBVPG 5195 and 5870: 5 days; DBVPG 5319: 6; DBVPG 4794: 8; DBVPG 5411: 9; and DBVPG 10019: 10)

**Fig. 5:**
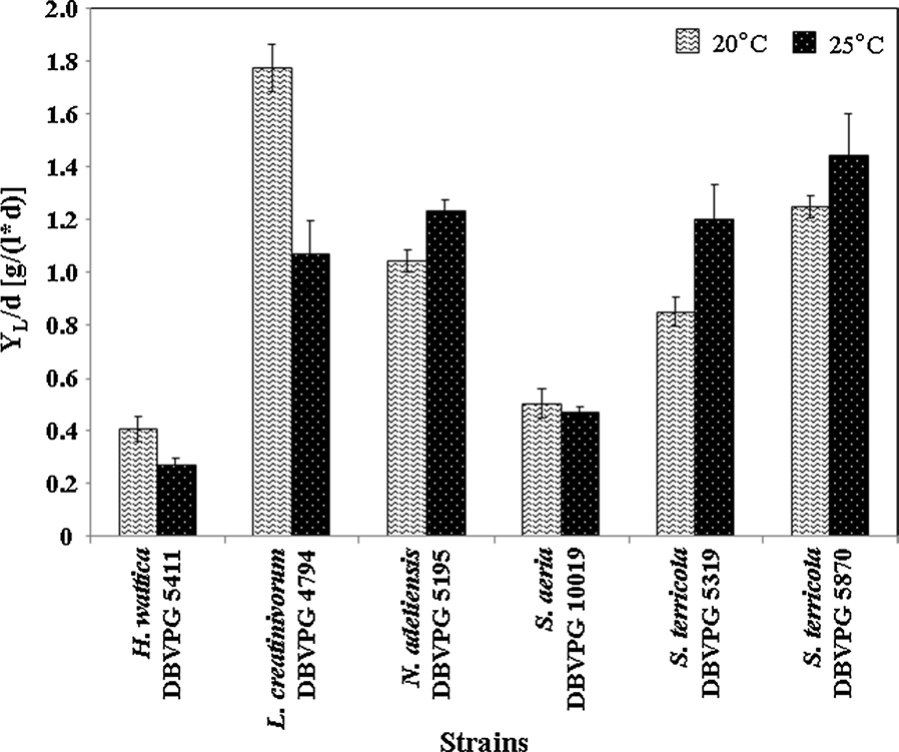
Total lipid yield daily productivity of selected yeast strains grown at 20 and 25 °C. Strains: *Holtermanniella wattica* DBVPG 5411, *Leucosporidium creatinivorum* DBVPG 4794, *Naganishia adeliensis* DBVPG 5195, *Solicoccozyma aeria* DBVPG 10019, *Solicoccozyma terricola* DBVPG 5319 and DBVPG 5870; Y_L_/d = total lipid yield daily productivity [g/(l*d)]. Media: GMY; temperature: 20 and 25 °C; incubation time is reported in the captions of Figs. 3 and 4

Y_L_, DW, Y_L_/DW, and Y_L_/Glu of the strains *H. wattica* DBVPG 5411, *L. creatinivorum* DBVPG 4794, *N. adeliensis* DBVPG 5195, *S. aeria* DBVPG 10019, and *S. terricola* DBVPG 5319 and DBVPG 5870 grown at two different temperatures (20–25 °C) are reported in Figs. 3 and 4, respectively. Overall, *L. creatinivorum* DBVPG 4794 exhibited a superior lipogenic aptitude: Y_L_ = 7.09 and 8.54 at 20 and 25 °C, respectively; Y_L_/DW > 70% at 20 °C; Y_L_/Glu = 21.42% at 25 °C (Figs. 3, 4). The Y_L_/d of this strain resulted around 1.8 g/(l*d) at 20 °C (Fig. 5). *N. adeliensis*, *S. aeria*, and *S. terricola* strains exhibited Y_L_ and Y_L_/DW comparable (in a few cases even higher) to those reported in literature [11, 12]. The time courses of lipid production (Y_L_, DW, Y_L_/DW, and Y_L_/Glu) of *L. creatinivorum* DBVPG 4794 (grown at 20 °C), *N. adeliensis* DBVPG 5195, and *S. terricola* DBVPG 5870 (both at 25 °C) are reported in Additional file 6: Figure S5.

A separate mention needs to be addressed for the two strains of the species *H. wattica* and *L. creatinivorum*. Both species were not previously regarded as oleaginous yeasts. In particular, the *H. wattica* has never been studied for its lipogenic aptitude, while a strain of *L. creatinivorum* has been taken into consideration recently, but its low lipogenic performances did not allow considering it as “oleaginous” [39]. The present study is the first to reveal the potential of these two species, in particular *L. creatinivorum*, as novel oleaginous yeasts.

The lipogenic ability of *L. creatinivorum* DBVPG 4794, *N. adeliensis* DBVPG 5195, and *S. terricola* DBVPG 5870 was compared with that of the bench marker-type strains of *S. cerevisiae* and *Y. lipolytica*. Interestingly, the last two species exhibited a lower lipogenic ability (*S. cerevisiae*: Y_L_ = 0.52 ± 0.1 and 0.57 ± 0.03 g/l; Y_L_/DW = 23.1 ± 0.4 and 15.5 ± 0.9%, at 20 and 25 °C, respectively; *Y. lipolytica*: Y_L_ = 2.16 ± 0.1 and 2.64 ± 0.1 g/l; Y_L_/DW = 25.1 ± 1.0 and 28.0 ± 0.5%, at 20 and 25 °C).

The fatty acid (FA) profiles of the six strains grown at 20 and 25 °C are reported in Table 1. Overall, the prevalent fatty acids were palmitic (hexadecanoic acid = C16:0), stearic (octadecanoic acid = C18:0), oleic [(9E9Z)-octadec-9-enoic acid = Δ9C18:1], and linoleic [(9Z,12Z)-9,12-octadecadienoic acid = Δ9,12C18:2] acids, with only lesser amounts of palmitoleic [(9Z)-hexadec-9-enoic acid = Δ9C16:1), margaric (heptadecanoic acid = C17:0), arachic (eicosanoic acid = C20:0), behenic (docosanoic acid = C22:0), and lignoceric (tetracosanoic acid = C24:0) acids. Surprisingly, no presence of linolenic acid [(9Z,12Z,15Z)-9,12,15-octadecatrienoic acid = Δ9,12,15C18:3] was found when strains were grown on glucose.

**Table 1:** Fatty acid profiles of *Holtermanniella wattica* DBVPG 5411, *Leucosporidium creatinivorum* DBVPG 4794, *Naganishia adeliensis* DBVPG 5195, *Solicoccozyma aeria* DBVPG 10019, *Solicoccozyma terricola* DBVPG 5319 and DBVPG 5870 grown at 20–25 °C

Strain	C16:0 (%)	Δ9C16:1 (%)	C17:0 (%)	C18:0 (%)	Δ9C18:1 (%)	Δ9,12C18:2 (%)	C20:0 (%)	C22:0 (%)	C24:0 (%)	Saturated fatty acids (%)	Unsaturated fatty acids (%)	UI
20 °C												
*H. wattica* DBVPG 5411	21.39 ± 2.12	0	0.56 ± 0.10	14.32 ± 0.11	44.17 ± 3.05	17.63 ± 1.73	1.19 ± 0.26	0.37 ± 0.65	0.35 ± 0.61	38.19	61.80	0.88
*L. creatinivorum* DBVPG 4794	23.79 ± 3.81	0.31 ± 0.53	0.04 ± 0.07	5.17 ± 0.43	62.42 ± 3.56	8.14 ± 2.77	0.14 ± 0.25	0	0	29.14	70.87	0.79
*N. adeliensis* DBVPG 5195	19.21 ± 2.00	0.11 ± 0.10	0.08 ± 0.07	5.67 ± 0.59	60.86 ± 3.92	13.42 ± 0.76	0.48 ± 0.44	0.13 ± 0.11	0.05 ± 0.08	25.62	74.39	0.88
*S. aeria* DBVPG 10019	23.28 ± 1.12	1.15 ± 0.02	0.18 ± 0.01	4.66 ± 0.38	53.2 ± 0.64	16.62 ± 0	0.37 ± 0.08	0	0.47 ± 0.12	28.96	70.97	0.88
*S. terricola* DBVPG 5319	26.11 ± 2.38	0.98 ± 0.09	0.18 ± 0.16	7.29 ± 0.63	57.71 ± 0.88	7.56 ± 0.94	0.18 ± 0.16	0	0	33.76	66.25	0.74
*S. terricola* DBVPG 5870	26.64 ± 1.77	1.17 ± 0.10	0.05 ± 0.08	7.02 ± 0.42	55.98 ± 2.79	8.81 ± 0.81	0.22 ± 0.19	0	0.11 ± 0.18	34.04	65.96	0.75
25 °C												
*H. wattica* DBVPG 5411	18.61 ± 2.07	0	0.41 ± 0.36	14.06 ± 0.56	56.16 ± 1.98	8.64 ± 0.76	0.88 ± 0.13	0.55 ± 0.55	0.70 ± 0.71	35.21	64.80	0.73
*L. creatinivorum* DBVPG 4794	20.94 ± 0.96	0.39 ± 0.10	0.05 ± 0.08	8.02 ± 1.24	61.09 ± 3.46	8.95 ± 0.97	0.46 ± 0.18	0.10 ± 0.17	0	29.57	70.43	0.79
*N. adeliensis* DBVPG 5195	20.90 ± 1.96	0.11 ± 0.09	0.09 ± 0.10	6.83 ± 1.74	64.41 ± 3.62	6.98 ± 1.06	0.62 ± 0.15	0.07 ± 0.12	0	27.70	71.50	0.78
*S. aeria* DBVPG 10019	20.67 ± 2.15	0.20 ± 0.35	0.08 ± 0.13	4.89 ± 0.52	63.85 ± 4.01	10.08 ± 1.77	0.09 ± 0.16	0	0.14 ± 0.24	25.87	74.12	0.84
*S. terricola* DBVPG 5319	26.17 ± 0.65	0.41 ± 0.42	0	6.31 ± 0.48	63.12 ± 2.39	3.98 ± 1.02	0	0	0	35.21	64.80	0.71
*S. terricola* DBVPG 5870	31.39 ± 2.08	0.85 ± 0.09	0	8.08 ± 1.06	53.60 ± 1.45	5.98 ± 1.07	0.10 ± 0.17	0	0	39.57	60.43	0.66

*C16:0* palmitic acid (hexadecanoic acid), *Δ9C16:1* palmitoleic acid [(9Z)-hexadec-9-enoic acid], *C17:0* margaric acid (heptadecanoic acid), *C18:0* stearic acid (octadecanoic acid), *Δ9C18:1* oleic acid [(9E)-octadec-9-enoic acid], *Δ9,12C18:2* linoleic acid [(9Z,12Z)-9,12-octadecadienoic acid], *C20:0* arachic acid (eicosanoic acid), *C22:0* behenic acid (docosanoic acid), *C24:0* lignoceric acid (tetracosanoic acid). *UI* Unsaturation Index, calculated as reported in paragraph 2.8

The amount of oleic acid was always over 50% for all strains grown at both temperatures, with the sole exception of *H. wattica* DBVPG 5411, whose FA profile included about 44% of oleic acid at 20 °C. In addition, the same strain exhibited a significantly (*P* < 0.05) higher concentration of stearic acid (about 14%) at both temperatures (Table 1). Current literature reports that microbial synthesis of lipids involves the initial formation of C16 or C18 saturated fatty acids that can be modified through a sequential series of reactions catalyzed by some desaturases and elongases, which form a number of both MUFAs and PUFAs. In this trend, oleic acid is the principal FA, accumulated in percentages close to 60% of total lipids, while the linoleic acid is found to be the second. However, different environmental conditions, especially the growth at different temperatures, can play a key role in varying the lipogenic aptitude and in changing the FA profiles, although this trend is not generally followed by all species [15, 48–50]. A quite variable FA profile was observed when the six strains were grown at the two tested temperatures. The decrease from 25 to 20 °C gave a significant (*P* < 0.05) increase of the concentration of linoleic acid for *H. wattica* DBVPG 5411, *N. adeliensis* DBVPG 5195, *S. aeria* DBVPG 10019, and both strains of *S. terricola*, in parallel with an increase of unsaturation index (UI). On the contrary, no significant (*P* < 0.05) variations were observed in FA profile of *L. creatinivorum* DBVPG 4794 at the two temperatures (Table 1).

**Fig. 6:**
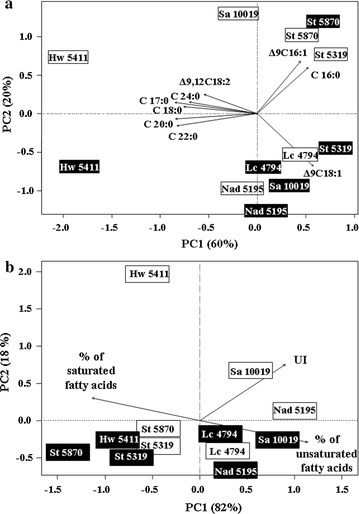
PCA of selected yeast strains as a function of their fatty acid profiles. Strains: *Holtermanniella wattica* DBVPG 5411 (Hw 5411), *Leucosporidium creatinivorum* DBVPG 4794 (Lc 4794), *Naganishia adeliensis* DBVPG 5195 (Na 5195), *Solicoccozyma aeria* DBVPG 10019 (Sa 10019), *Solicoccozyma terricola* DBVPG 5319 (St 5319) and DBVPG 5870 (St 5870) grown at 20 (*white rectangles*) and 25 °C (*black rectangles*). **a** *C16:0* palmitic acid (hexadecanoic acid); *Δ9C16:1* palmitoleic acid [(9Z)-hexadec-9-enoic acid]; *C17:0* margaric acid (heptadecanoic acid); *C18:0* stearic acid (octadecanoic acid); *Δ9C18:1* oleic acid [(9E)-octadec-9-enoic acid]; *Δ9,12C18:2* linoleic acid [(9Z,12Z)-9,12-octadecadienoic acid]; *C20:0* arachic acid (eicosanoic acid); *C22:0* behenic acid (docosanoic acid); *C24:0* lignoceric acid (tetracosanoic acid). **b** *UI* Unsaturation Index, calculated as reported in the text. Media: GMY; temperature: 20 and 25 °C; incubation time is reported in the captions of Figs. 3 and 4

PCA was used to ordinate the above six strains based on their fatty acid profiles observed at both 20 and 25 °C (Fig. 6a). Strains were clustered into three groups: the first included only *H. wattica* DBVPG 5411 (grown at both 20 and 25 °C) and showed a high (above average) concentration in stearic, linoleic, margaric, arachic, behenic, and lignoceric acids; the second group included *S. terricola* DBVPG 5870 (grown at both 20 and 25 °C), and *S. aeria* DBVPG 10019 and *S. terricola* DBVPG 5319 (both grown only at 20 °C), which showed a high concentration in palmitic and palmitoleic acids; and the third group, characterized by a high concentration in oleic acid, included *L. creatinivorum* DBVPG 4794 and *N. adeliensis* DBVPG 5195 (both grown at 20 and 25 °C), and *S. aeria* DBVPG 10019 and *S. terricola* DBVPG 5319 (both grown only at 25 °C) (Fig. 6a). PCA was also used to ordinate the six strains based on their cumulative FA profiles (% of saturated and unsaturated fatty acids and UI) observed at both 20 and 25 °C. Strains were clustered into three groups: (i) the first, characterized by high (above average) values in the % of saturated fatty acids, included both strains of *S. terricola* (grown at both 20 and 25 °C) and *H. wattica* DBVPG 5411 (grown only at 25 °C); (ii) the second group included *L. creatinivorum* DBVPG 4794, *N. adeliensis* DBVPG 5195, and *S. aeria* DBVPG 10019 (all grown at both 20 and 25 °C) which showed high % of unsaturated fatty acids and UI, yet in variable extent depending on the different strains; and (iii) the third group included only *H. wattica* DBVPG 5411 (grown at 20 °C), which was only partially related to the % of saturated fatty acids and UI (Fig. 6b).

### Lipid production on C3, C5, C6, and C12 carbon sources

Y_L_, DW, Y_L_/DW, the Y_L_/carbon source, and Y_L_/d of the strains *L. creatinivorum* DBVPG 4794, *N. adeliensis* DBVPG 5195, and *S. terricola* DBVPG 5870 grown on C3 (glycerol), C5 (xylose), C6 (galactose), and C12 (sucrose, cellobiose, and maltose) are reported in Table 2. All C3, C5, C6, and C12 carbohydrates were selected for the study because they are representative of common sustainable biological waste, i.e., glycerol results from trans-esterification process for biodiesel production, xylose from hydrolysis of hemicellulosic residues, galactose from hydrolysis of whey lactose, sucrose from molasses, maltose from hydrolysis of wort from brewing processes, and cellobiose from lignocellulosic residues [11, 12, 51].

**Table 2:** Lipid and biomass yields of *Leucosporidium creatinivorum* DBVPG 4794 (at 20 °C), *Naganishia adeliensis* DBVPG 5195 and *Solicoccozyma terricola* DBVPG 5870 (both at 25 °C) on different C sources

Carbon source	Strains		
	*L. creatinivorum* DBVPG 4794	*N. adeliensis* DBVPG 5195	*S. terricola* DBVPG 5870
C3			
Glycerol			
Y_L_ (g/l)	7.3 ± 0.4	No growth	No growth
DW (g/l)	14.1 ± 2.0		
Y_L_/DW (%)	52 ± 4.9		
Y_L_/Gly (%)	18 ± 0.4		
Y_L_/d [g/(l*d)]	0.38 ± 0.4		
C5			
Xylose			
Y_L_ (g/l)	5.0 ± 0.5	4.3 ± 0.1	6.8 ± 0.4
DW (g/l)	10.5 ± 0.2	10.1 ± 0.2	11.9 ± 0.3
Y_L_/DW (%)	48 ± 4	42 ± 2	57 ± 3
Y_L_/Xyl (%)	13 ± 0.5	11 ± 0.1	17 ± 0.4
Y_L_/d [g/(l*d)]	0.38 ± 0.5	0.33 ± 0.1	0.97 ± 0.4
C6			
Galactose			
Y_L_ (g/l)	No growth	No growth	7.9 ± 0.6
DW (g/l)			13.6 ± 1.1
Y_L_/DW (%)			59 ± 8.7
Y_L_/Gal (%)			20 ± 0.6
Y_L_/d [g/(l*d)]			1.58 ± 0.6
C12			
Sucrose			
Y_L_ (g/l)	6.5 ± 0.6	6 ± 0.6	6.9 ± 0.8
DW (g/l)	15.6 ± 2.4	14 ± 1.2	13.9 ± 0.4
Y_L_/DW (%)	42 ± 4	43 ± 2	50 ± 5.5
Y_L_/Suc (%)	17 ± 0.6	16 ± 0.6	18 ± 0.8
Y_L_/d [g/(l*d)]	1.63 ± 0.6	1.2 ± 0.6	1.73 ± 0.8
Maltose			
Y_L_ (g/l)	4.8 ± 0.7	6.2 ± 0.4	7.9 ± 0.6
DW (g/l)	10.4 ± 1.1	12 ± 1.4	14.1 ± 0.2
Y_L_/DW (%)	46 ± 4.2	52 ± 5.3	56 ± 4.9
Y_L_/Mal (%)	13 ± 0.7	16 ± 0.4	21 ± 0.6
Y_L_/d [g/(l*d)]	0.69 ± 0.7	0.56 ± 0.4	1.58 ± 0.6
Cellobiose			
Y_L_ (g/l)	No growth	6.4 ± 0.4	5.9 ± 1.1
DW (g/l)		12.9 ± 1.8	11.9 ± 1.4
Y_L_/DW (%)		50 ± 7.4	50 ± 7.4
Y_L_/Cel (%)		17 ± 0.4	16 ± 1.1
Y_L_/d [g/(l*d)]		0.43 ± 0.4	1.06 ± 1.1

*Y*_*L*_ total lipid yield, *DW* cell dry weight, *Y*_*L*_ */DW* % of intracellular lipids on DW, *Y*_*L*_ */Gly* % of lipid produced in 100 g of glycerol, *Y*_*L*_ */Xyl* % of lipid produced in 100 g of xylose, *Y*_*L*_ */Gal* % of lipid produced in 100 g of galactose, *Y*_*L*_ */Suc* % of lipid produced in 100 g of sucrose, *Y*_*L*_ */Mal* % of lipid produced in 100 g of maltose, *Y*_*L*_ */Cel* % of lipid produced in 100 g of cellobiose, *Y*_*L*_ */d* total lipid yield daily productivity

None of the carbon sources supported growth and lipid production by all the three above-mentioned strains. In addition, the use of different carbon sources considerably affected Y_L_, DW, Y_L_/DW, Y_L_/carbon source, Y_L_/d (Table 2), and FA profile (Table 3), in agreement with current literature that reports that the type of carbon source has a great influence over growth rate, lipid yields, and fatty acids composition [51, 52].

**Table 3:** Fatty acid profiles of *Leucosporidium creatinivorum* DBVPG 4794 (at 20 °C), *Naganishia adeliensis* DBVPG 5195 and *Solicoccozyma terricola* DBVPG 5870 (both at 25 °C) on different C sources

Strain	Carbon source	C 16:0 (%)	Δ9,C16:1 (%)	C 17:0 (%)	C 18:0 (%)	Δ9,C18:1 (%)	Δ9,12, C18:2 (%)	Δ9,12,15 C18:3 (%)	C 20:0 (%)	C 22:0 (%)	C 24:0 (%)	Saturated fatty acids (%)	Unsaturated fatty acids (%)	UI
*L. creatinivorum* DBVPG 4794	Glycerol	33.85 ± 5.71	0.38 ± 0.17	0.02 ± 0.03	9.42 ± 2.61	44.61 ± 0.12	11.23 ± 3.05	0.34 ± 0.06	0.14 ± 0.20	0	0	43.43	56.57	0.68
	Xylose	18.83 ± 0.75	0.11 ± 0.10	0.03 ± 0.05	13.35 ± 0.83	56.89 ± 1.05	8.27 ± 0.75	1.26 ± 0.19	0.63 ± 0.10	0.48 ± 0.05	0.16 ± 0.15	33.48	66.52	0.77
	Maltose	22.48 ± 1.08	0.30 ± 0.05	0.09 ± 0.01	5.31 ± 0.55	58.79 ± 1.23	9.15 ± 0.59	3.27 ± 1.13	0.32 ± 0.10	0.24 ± 0.05	0.05 ± 0.03	28.49	71.51	0.87
	Sucrose	21.79 ± 2.77	0.56 ± 0.32	0.09 ± 0.04	6.29 ± 1.85	61.16 ± 2.32	7.91 ± 2.00	1.61 ± 0.33	0.40 ± 0.08	0.18 ± 0.16	0.02 ± 0.03	28.77	71.23	0.82
*N. adeliensis* DBVPG 5195	Xylose	20.99 ± 1.23	0.05 ± 0.05	0.03 ± 0.04	6.33 ± 0.09	56.99 ± 1.86	15.13 ± 2.41	0.22 ± 0.09	0.23 ± 0.10	0	0.04 ± 0.07	27.61	72.39	0.88
	Cellobiose	22.86 ± 1.99	0.03 ± 0.05	0	8.00 ± 0.56	52.79 ± 3.12	14.94 ± 2.39	0.73 ± 0.41	0.49 ± 0.28	0.16 ± 0.10	0	31.51	68.49	0.85
	Maltose	23.91 ± 0.68	0.11 ± 0.02	0.07 ± 0.02	6.18 ± 0.86	55.45 ± 3.67	13.46 ± 2.75	0.22 ± 0.07	0.42 ± 0.12	0.07 ± 0.07	0.09 ± 0.09	30.75	69.25	0.83
	Sucrose	25.02 ± 0.81	0.15 ± 0.02	0.09 ± 0.01	5.97 ± 0.65	58.17 ± 2.35	10.16 ± 2.53	0.25 ± 0.01	0.16 ± 0.03	0.02 ± 0.03	0.01 ± 0.01	31.26	68.74	0.79
*S. terricola* DBVPG 5870	Xylose	30.60 ± 2.40	0.82 ± 0.30	0.27 ± 0.07	13.26 ± 1.74	47.25 ± 1.61	7.24 ± 3.27	0	0.37 ± 0.11	0.07 ± 0.08	0.12 ± 0.20	44.69	55.31	0.63
	Galactose	30.37 ± 1.53	0.56 ± 0.44	0.14 ± 0.05	10.95 ± 1.32	53.06 ± 1.65	4.23 ± 1.07	0	0.31 ± 0.14	0.13 ± 0.08	0.25 ± 0.21	42.16	57.84	0.62
	Cellobiose	30.37 ± 1.36	0.40 ± 0.06	0.34 ± 0.05	22.83 ± 0.42	30.78 ± 0.51	13.62 ± 1.10	0.21 ± 0.03	0.64 ± 0.09	0.28 ± 0.06	0.53 ± 0.13	54.99	45.01	0.59
	Maltose	31.31 ± 1.27	0.87 ± 0.35	0.14 ± 0.04	10.74 ± 1.15	48.26 ± 2.61	7.91 ± 2.00	0	0.35 ± 0.13	0.13 ± 0.15	0.30 ± 0.20	42.96	57.04	0.65
	Sucrose	28.66 ± 4.33	1.11 ± 1.58	0.11 ± 0.02	11.44 ± 1.91	53.63 ± 2.69	4.78 ± 2.65	0	0.16 ± 0.14	0.05 ± 0.04	0.07 ± 0.06	40.48	59.52	0.64

*C16:0* palmitic acid (hexadecanoic acid), *Δ9C16:1* palmitoleic acid [(9Z)-hexadec-9-enoic acid], *C17:0* margaric acid (heptadecanoic acid), *C18:0* stearic acid (octadecanoic acid), *Δ9C18:1* oleic acid [(9E)-octadec-9-enoic acid], *Δ9,12C18:2* linoleic acid [(9Z,12Z)-9,12-octadecadienoic acid], *Δ9,12,15C18:3* linolenic acid [(9Z,12Z,15Z)-9,12,15-octadecatrienoic acid], *C20:0* arachic acid (eicosanoic acid), C22:0 behenic acid (docosanoic acid), *C24:0* lignoceric acid (tetracosanoic acid). *UI* Unsaturation Index, calculated as reported in paragraph 2.8

*Leucosporidium creatinivorum* DBVPG 4794 gave the maximum Y_L_ (7.3 g/l) at 20 °C on glycerol, while *N. adeliensis* DBVPG 5195 gave a Y_L_ around 6 g/l at 25 °C on sucrose, maltose, and cellobiose, and *S. terricola* DBVPG 5870 gave the maximum Y_L_ (7.9 g/l) at 25 °C on maltose and galactose. On the whole, all strains exhibited a Y_L_/DW around to 50% and a Y_L_/Gly below 20%. The highest Y_L_/d was found when strains were grown on sucrose (Table 2).

FA profiles of the strains *L. creatinivorum* DBVPG 4794, *N. adeliensis* DBVPG 5195, and *S. terricola* DBVPG 5870 grown on the C3, C5, C6, and C12 carbon sources are reported in Table 3. Palmitic, stearic, oleic, and linoleic acids were the prevalent FAs observed. The use of carbon sources different from glucose caused some significant (*P* < 0.05) changes in the FA profiles of both *L. creatinivorum* DBVPG 4794 and *S. terricola* DBVPG 5870, in agreement with previous studies reporting that differences in carbon and nitrogen sources greatly affect lipogenic aptitude of yeasts [4, 10–12, 14, 35, 36]. In detail, the growth of *L. creatinivorum* DBVPG 4794 on glycerol gave an increased concentration of palmitic acid over 30% of total lipids, only partially balanced by a decrease of oleic acid below to 50%. A decrease of the % of unsaturated fatty acids and UI was consequently observed (Table 3). Likewise, the growth of *S. terricola* DBVPG 5870 on cellobiose gave a lower concentration of oleic acid of about 30% of total lipids and a parallel higher concentration of both stearic and linoleic acids up to about 22.83 and 13.62%, respectively. As a result, a decrease of the % of unsaturated fatty acids (below 50%) and UI was observed (Table 3). Interestingly, the growth of the above three strains on C3, C5, C6, and C12 carbon sources stimulated the production of little amounts of linolenic acid (Table 3).

## Conclusions

A screening on a large set of yeasts isolated worldwide allowed the selection of a few lipid-overproducing strains. Variable lipid yields and fatty acid profiles were observed in dependence of both temperature and different carbon sources. The strain *L. creatinivorum* DBVPG 4794 exhibited the highest lipogenic performances. Further studies are under way to test the lipogenic aptitude of this strain on raw substrates (as carbon sources) of agricultural and industrial origin, and the subsequent scaling-up in fermenter on a laboratory scale.

## Additional files

**Additional file 1: Table S1.** Salient information on all yeasts used in this study. DBVPG accession number, species, isolation source, isolation Locality, optimal growth temperature, Phylum of yeast strains used in this study.

**Additional file 2: Figure S1.** Flow chart figure reporting the workflow of the study. A flow chart figure summarizing the workflow of the study.

**Additional file 3: Figure S2.** A few examples of micrographs of yeast strains before and during fluorescence emission. Increasing intracellular lipid yield evaluated by Nile Red before (**A**, **C**, **E** and **G**) and during fluorescence emission (B, D, F and H) photographed with a Wild MP 552 camera (Leica). **A** and **B** = *Debaryomyces hansenii* DBVPG 3326; C and D = *Zygosaccharomyces bisporus* DBVPG 3018; **E** and **F** = *Magnusiomyces capitatus* DBVPG 3250; **G** and **H** = Naganishia albida DBVPG 4919.

**Additional file 4: Figure S3.** High resolution images of NR staining of *Leucosporidium creatinivorum* DBVPG 4794 (incubated at 20 °C), *Naganishia adeliensis* DBVPG 5195 and *Solicoccozyma terricola* DBVPG 5870 (both at 25 °C). High resolution images of Nile Red staining of *Naganishia adeliensis* DBVPG 5195 (**A** and **B**, incubated at 25 °C), *Solicoccozyma terricola* DBVPG 5870 (**C** and **D**, 25 °C) and *Leucosporidium creatinivorum* DBVPG 4794 (E and F, 20 °C). Photographs were captured before (**A**, **C** and **E**) and during fluorescence emission (**B**, **D** and **F**) with an UV epifluorescence microscope Olympus BX53 (Olympus Co., Centre Valley, PA, USA) equipped with excitation filter BP470-500 and barrier filter BA515-560 and a XC50 camera (Olympus Co.).

**Additional file 5: Figure S4.** 2D scatterplot of EILY vs DW of 706 yeast strains studied. Estimated Intracellular Lipid Yield (EILY, calculated as reported in the text) and cell dry weight (DW) of the strains herein studied assessed by a Kruskal–Wallis test (*P* < 0.05). **A** comparison of EILY and DW in dependence of taxonomic position (Ascomycota vs Basidiomycota) of the 706 strains. Red symbols = ascomycetous strains; black symbols = basidiomycetous strains. **A** and **B** = centroids of ascomycetous and basidiomycetous strains, respectively. **B** comparison of EILY and DW in dependence of optimal growth temperature (psychrophiles vs psychrotolerant vs mesophiles) of 422 basidiomycetous strains. Red symbols = psychrophilic strains; blue symbols = psychrotolerant strains; black symbols = mesophilic strains. P, Pt and M = centroids of psychrophilic, psychrotolerant and mesophilic strains, respectively.

**Additional file 6: Figure S5.** Time course of lipid production of *Leucosporidium creatinivorum* DBVPG 4794 (grown at 20 °C), *Naganishia adeliensis* DBVPG 5195 and *Solicoccozyma terricola* DBVPG 5870 (both at 25 °C). Y_L_ = Total lipid yield; DW = cell dry weight; Y_L_/DW = total intracellular lipids on cell biomass; Y_L_/Glu = lipid coefficient.

## Abbreviations

TAGs: triacylglycerols
MUFAs: monounsaturated fatty acids
PUFAs: polyunsaturated fatty acids
OD_600_: optical density measured at 600 nm
EILY: estimated intracellular lipid yield
IEF: intensity of emitted fluorescence
TLPA: total lipid particle area
CA: cell area
GC-MS: gas chromatography-mass spectrometry
ANOVA: analysis of variance
Y_L_: total lipid yield
DW: cell dry weight
Y_L_/DW: % of intracellular lipids on DW
Y_L_/d: total lipid yield daily productivity
PCA: principal components analysis
Y_L_/Glu: % of lipid produced in 100 g of glucose
Y_L_/Gly: % of lipid produced in 100 g of glycerol
Y_L_/Xyl: % of lipid produced in 100 g of xylose
Y_L_/Gal: % of lipid produced in 100 g of galactose
Y_L_/Suc: % of lipid produced in 100 g of sucrose
Y_L_/Mal: % of lipid produced in 100 g of maltose
Y_L_/Cel: % of lipid produced in 100 g of cellobiose
C16:0: palmitic acid (hexadecanoic acid)
Δ9C16:1: palmitoleic acid [(9Z)-hexadec-9-enoic acid]
C17:0: margaric acid (heptadecanoic acid)
C18:0: stearic acid (octadecanoic acid)
Δ9C18:1: oleic acid [(9E)-octadec-9-enoic acid]
Δ9,12C18:2: linoleic acid [(9Z,12Z)-9,12-octadecadienoic acid]
C20:0: arachic acid (eicosanoic acid)
C22:0: behenic acid (docosanoic acid)
C24:0: lignoceric acid (tetracosanoic acid)
UI: Unsaturation Index, calculated as reported in the text
