# Supplementary material for: Study of Holtermanniella wattica, Leucosporidium creatinivorum, Naganishia adeliensis, Solicoccozyma aeria, and Solicoccozyma terricola for their lipogenic aptitude from different carbon sources
Source: Biotechnol Biofuels. 2016 Nov 28;9:259. doi: 10.1186/s13068-016-0672-1 (PMC5126845; doi:10.1186/s13068-016-0672-1)
Supplement: Supplementary file 1 — Additional file 1: Table S1. Salient information on all yeasts used in this study. DBVPG accession number, species, isolation source, isolation Locality, optimal growth temperature, Phylum of yeast strains used in this study. [file 13068_2016_672_MOESM1_ESM.pdf]

Additional file 1

Salient information on all yeasts used in this study.

DBVPG accession number	Species	Isolation source	Isolation Locality	Optimal growth temperature	Phylum
4325	*Candida catenulata*	frozen mushrooms	Italy	25°C	Ascomycota
3747	*Candida intermedia*	artichokes hearts under oil	S. Benedetto del Tronto, Italy	25°C	Ascomycota
3186; 3187	*Candida magnoliae*	flowers	Venarotta, Ascoli Piceno, Italy	25°C	Ascomycota
3188	*Candida magnoliae*	flowers	Perugia, Italy	25°C	Ascomycota
3834	*Candida magnoliae*	fodder yeast	Italy	25°C	Ascomycota
4327; 4409	*Candida norvegica*	sea water	Italy	25°C	Ascomycota
4624; 4636	*Candida norvegica*	soil at 850 m alt., 2 cm depth	Bromide Oklahoma, USA	25°C	Ascomycota
4467	*Candida oleophila*	mandarine orange	Italy	25°C	Ascomycota
4547	*Candida oleophila*	immature fruit of *Rhipsalis cassutha*, Cactaceae	Botanical garden, Rio de Janiero, Brazil	25°C	Ascomycota
4612	*Candida pararugosa*	sea water	Italy	25°C	Ascomycota
4660 - 4664	*Candida saitoana*	epicarp of black truffle, *Tuber melanosporum*	Fabriano, Ancona, Italy	25°C	Ascomycota

		Vitt.			
4856	*Candida saitoana*	cavern soil	Grotta del Vento, Marche, Italy	25°C	Ascomycota
4951	*Candida saitoana*	commercial compost, end of process, 3.5 months ca	Pietramelina, Perugia, Italy	25°C	Ascomycota
3744; 3745	*Candida sake*	soil	Bottenangen, Sweden	25°C	Ascomycota
4625	*Candida sake*	soil at 850 m alt., 2 cm depth	Bromide Oklahoma, USA	25°C	Ascomycota
4882; 4883; 4885	*Candida sake*	surface of Tuber mescutericum	Italy	25°C	Ascomycota
4648; 4649	*Candida santamariae*	soil around *Corylus avellana* in a truffle field, Tuber aestivum	Volperino, Perugia, Italy	25°C	Ascomycota
5182	*Candida santamariae*	supraglacial sediments	Calderone glacier, Gran Sasso, Abruzzo, Italy	25°C	Ascomycota
5397 - 5399	*Candida santamariae*	morainic soil	Miage glacier, Mont Blanc, Italian Alps, Italy	25°C	Ascomycota
3724 - 3727	*Candida vartiovaarae*	soil	Viik, Helsinki, Finland	25°C	Ascomycota
3414; 3415	*Candida versatilis*	milk	Italy	25°C	Ascomycota
3512	*Candida versatilis*			25°C	Ascomycota

3731	*Candida versatilis*	ensiled olive husks	Perugia, Italy	25°C	Ascomycota
4853	*Candida versatilis*	cavern soil	Grotta del Vento, Marche, Italy	25°C	Ascomycota
3952; 3953	*Candida zeylanoides*	natural rennet	Italy	25°C	Ascomycota
4137 - 4140	*Candida zeylanoides*	grape must	Slovenia	25°C	Ascomycota
3417	*Citeromyces matritensis*	milk	Perugia, Italy	25°C	Ascomycota
7088; 7089	*Clavispora opuntiae*	Opuntia sp.	Los Pirpinitos, Argentina	25°C	Ascomycota
3177; 3420	*Cyberlindnera jadinii*	flowers	Venarotta, Ascoli Piceno, Italy	25°C	Ascomycota
3201; 3202; 3738	*Cyberlindnera jadinii*			25°C	Ascomycota
3344	*Cyberlindnera jadinii*		Etna, Sicily, Italy	25°C	Ascomycota
3431; 3432	*Cyberlindnera jadinii*		ex Yugoslavia	25°C	Ascomycota
3709; 3710	*Cyberlindnera jadinii*		Fumane, Verona, Italy	25°C	Ascomycota
3837	*Cyberlindnera jadinii*	flowers		25°C	Ascomycota
3124; 3125 - 3130	*Cyberlindnera saturnus*	soil	The Netherlands	25°C	Ascomycota
3671	*Cyberlindnera saturnus*	soil	Hyytiala, Finland	25°C	Ascomycota

4561	*Cyberlindnera saturnus*	flower of Compositae	Pao da Fome, Pedra Branca, Brazil	25°C	Ascomycota
4889	*Cyberlindnera saturnus*	surface of *Tuber imelaia*	Italy	25°C	Ascomycota
2507; 3485	*Debaryomyces hansenii*			25°C	Ascomycota
3326; 3328 - 3332	*Debaryomyces hansenii*	Limburger cheese	Wageningen, The Netherlands	25°C	Ascomycota
3410	*Debaryomyces hansenii*		ex Yugoslavia	25°C	Ascomycota
3419	*Debaryomyces hansenii*	milk	Italy	25°C	Ascomycota
3682 - 3685; 3687; 3688	*Debaryomyces hansenii*	soil	Viik, Helsinki, Finland	25°C	Ascomycota
3978; 3979	*Debaryomyces hansenii*	caverns	Italy	25°C	Ascomycota
4276	*Debaryomyces hansenii*	human oral cavity	Perugia, Italy	25°C	Ascomycota
3145 - 3147	*Debaryomyces hansenii*	sausages	Italy	25°C	Ascomycota
3241 - 3246	*Geotrichum candidum*	Limburger cheese	Wageningen, The Netherlands	25°C	Ascomycota
4350	*Geotrichum candidum*	sea water	Italy	25°C	Ascomycota
3247; 3249; 3250	*Magnusiomyces capitatus*	Limburger cheese	The Netherlands	25°C	Ascomycota
4541	*Hyphopichia burtonii*	flower of *Miconia* sp., Milistomataceae	Angra dos Reis, Brazil	25°C	Ascomycota

6309; 6310	*Kazachstania africana*			25°C	Ascomycota
3191 - 3193	*Kazachstania exigua*	Limburger cheese	Maastricht, The Netherlands	25°C	Ascomycota
4384; 4385	*Kazachstania exigua*	sea water	Italy	25°C	Ascomycota
3423	*Kluyveromyces lactis*	milk	Italy	25°C	Ascomycota
3882; 3912	*Kluyveromyces lactis*	phlegm of a tuberculosis patient	Perugia, Italy	25°C	Ascomycota
6030	*Kluyveromyces lactis*	sputum	Spain	25°C	Ascomycota
4245	*Kluyveromyces lactis*	fodder additive "Zymoyeast"	Torino, Italy	25°C	Ascomycota
3084; 3211	*Kluyveromyces marxianus*			25°C	Ascomycota
3391; 3392	*Kluyveromyces marxianus*	pharnyx mucous	Perugia, Italy	25°C	Ascomycota
3436	*Kluyveromyces marxianus*	milk	Italy	25°C	Ascomycota
3748	*Kluyveromyces marxianus*	mozzarella cheese	Perugia, Italy	25°C	Ascomycota
3925 - 3929; 3939 - 3941	*Kluyveromyces marxianus*	natural rennet	Italy	25°C	Ascomycota
4241; 4243; 4246	*Kluyveromyces marxianus*	food additive	Torino, Italy	25°C	Ascomycota
3362 - 3368	*Kodamaea ohmeri*	flowers	Perugia, Italy	25°C	Ascomycota

3437	*Kodamaea ohmeri*	milk	Perugia, Italy	25°C	Ascomycota
3438	*Kodamaea ohmeri*		Capoterra, Sardinia, Italy	25°C	Ascomycota
3439	*Kodamaea ohmeri*	grape must	Greece	25°C	Ascomycota
3227; 3228	*Kregervanrija fluxuum*	soil	Italy	25°C	Ascomycota
3229 - 3232	*Kregervanrija fluxuum*			25°C	Ascomycota
3761	*Kregervanrija fluxuum*	artichokes hearts under oil	Montalto di Castro, Viterbo, Italy	25°C	Ascomycota
3765	*Kregervanrija fluxuum*	ensiled olive and tomato residues	Italy	25°C	Ascomycota
4048	*Kregervanrija fluxuum*	grape must	ex Yugoslavia	25°C	Ascomycota
4049	*Kregervanrija fluxuum*	grape must	Verona, Italy	25°C	Ascomycota
4141 - 4146	*Kregervanrija fluxuum*	grape must	Slovenia	25°C	Ascomycota
3343	*Kuraishia capsulata*	flowers	Perugia, Italy	25°C	Ascomycota
3674 - 3681; 3721 - 3723	*Kuraishia capsulata*	soil	Hyytiala, Finland	25°C	Ascomycota
3103 - 3107	*Lachancea kluyveri*	soil	Italy	25°C	Ascomycota
3108 - 3110	*Lachancea kluyveri*	soil	The Netherlands	25°C	Ascomycota

3452 - 3454	*Lachancea kluyveri*	soil	Ultuna, Sweden	25°C	Ascomycota
3470; 3785	*Lachancea kluyveri*	soil	Okana, Spain	25°C	Ascomycota
4003 - 4005	*Lachancea kluyveri*	caverns	Italy	25°C	Ascomycota
3174	*Lachancea thermotolerans*			25°C	Ascomycota
3785; 3395	*Lachancea thermotolerans*		Delianova, Sardinia, Italy	25°C	Ascomycota
3418	*Lachancea thermotolerans*	milk	Italy	25°C	Ascomycota
3464; 3465	*Lachancea thermotolerans*	grapes	Valdepenas, La Mancha, Spain	25°C	Ascomycota
3466 - 3468	*Lachancea thermotolerans*	grapes	La Encomienda, La Mancha, Spain	25°C	Ascomycota
3469	*Lachancea thermotolerans*	grapes	Manzanares, La Mancha, Spain	25°C	Ascomycota
3597; 3598	*Lachancea thermotolerans*		Illasi, Verona, Italy	25°C	Ascomycota
3599	*Lachancea thermotolerans*		Soave, Verona, Italy	25°C	Ascomycota
3600	*Lachancea thermotolerans*		Aguilar, Cordoba, Spain	25°C	Ascomycota
3601 - 3603	*Lachancea thermotolerans*		Moriles Altos, Cordoba, Spain	25°C	Ascomycota
3604; 3605	*Lachancea thermotolerans*		Sierra Montilla, Cordoba, Spain	25°C	Ascomycota

6193; 6637	*Lipomyces starkeyi*	soil	USA	25°C	Ascomycota
3247; 3249 - 3253	*Magnusiomyces capitatus*	Limburger cheese	The Netherlands	25°C	Ascomycota
3158	*Magnusiomyces magnusii*	sausages	Italy	25°C	Ascomycota
3009	*Metschnikowia pulcherrima*	grape must	Umbria, Italy	25°C	Ascomycota
3015	*Metschnikowia pulcherrima*	grape must	Chianti, Italy	25°C	Ascomycota
3032 - 3034	*Metschnikowia pulcherrima*	grape must	Lucera, Italy	25°C	Ascomycota
3041	*Metschnikowia pulcherrima*	cherris	Italy	25°C	Ascomycota
3042	*Metschnikowia pulcherrima*	sour black cherries	Italy	25°C	Ascomycota
3043	*Metschnikowia pulcherrima*	grape must	Isola d'Elba, Toscana, Italy	25°C	Ascomycota
3086	*Metschnikowia pulcherrima*	grape must	Trentino, Italy	25°C	Ascomycota
3356 - 3359	*Metschnikowia reukaufii*	flowers	Venarotta, Ascoli Piceno, Italy	25°C	Ascomycota
3838; 3839	*Metschnikowia reukaufii*	flowers	Italy	25°C	Ascomycota
3630 - 3633; 3635	*Millerozyma farinosa*	soil	Teisko, Finland	25°C	Ascomycota
4018	*Millerozyma farinosa*	wine	Italy	25°C	Ascomycota

3728 - 3730; 3732 - 3735	*Nakazawaea wickerhamii*	cavern soil	Grotta del Vento, Marche, Italy	25°C	Ascomycota
3669; 3670; 3672; 3673	*Ogataea minuta*	soil	Hyytiala, Finland	25°C	Ascomycota
4516 - 4518	*Ogataea polimorpha*			25°C	Ascomycota
3807 - 3809	*Pichia fermentans*	soil		25°C	Ascomycota
4359; 4360	*Pichia fermentans*	frozen fava beans	Italy	25°C	Ascomycota
4361 - 4363	*Pichia fermentans*	sea water	Italy	25°C	Ascomycota
3628; 3629	*Pichia fermentans*	soil	Teisko, Finland	25°C	Ascomycota
3506	*Priceomyces carsonii*			25°C	Ascomycota
4526; 4527	*Saturnispora silvae*	Vriesea friburgensis	Tijuca forest, Brazil	25°C	Ascomycota
4549	*Schwanniomyces etchellsii*	exudate of *Eriobotrya japonica*, Rosaceae	Tijuca forest, Brazil	25°C	Ascomycota
3509; 3510	*Schwanniomyces occidentalis*	soil	Aranjuez, Spain	25°C	Ascomycota
4916	*Schwanniomyces occidentalis*	root of *Quercus pubescens* in a truffle field, *Tuber aestivum*	Volperino, Perugia, Italy	25°C	Ascomycota
3690 - 3694; 3696	*Schwanniomyces polymorphus*	soil	Teisko, Finland	25°C	Ascomycota

3697; 3699 - 3701	*Schwanniomyces polymorphus*	soil	Hyytiala, Finland	25°C	Ascomycota
4944	*Schwanniomyces polymorphus*	*Tuber aestivum* associated with *Corylus avellana*	Volperino, Perugia, Italy	25°C	Ascomycota
4122; 4124; 4126	*Starmerella bombicola*	grape must	Slovenia	25°C	Ascomycota
3711	*Starmerella bombicola*		Ruedos Aguilar, Cordoba, Spain	25°C	Ascomycota
3016	*Zygosaccharomyces bisporus*		Colli Romani, Lazio, Italy	25°C	Ascomycota
3018	*Zygosaccharomyces bisporus*			25°C	Ascomycota
4372; 4373	*Buckleyzyma aurantiaca*	sea water	Italy	25°C	Basidiomycota
4606; 4608	*Buckleyzyma aurantiaca*	flower of Compositae	Botanical garden, Rio de Janiero, Brazil	25°C	Basidiomycota
4607	*Buckleyzyma aurantiaca*	*Trigona* sp., Apidae	Botanical garden, Rio de Janiero, Brazil	25°C	Basidiomycota
4609; 4610	*Buckleyzyma aurantiaca*	flower of *Piper aduncum*	Botanical garden, Rio de Janiero, Brazil	25°C	Basidiomycota
4320; 4321	*Bullera alba*	sea water	Italy	25°C	Basidiomycota
4614	*Bullera alba*	soil at 850 m alt., 2 cm depth	Bromide Oklahoma, USA	25°C	Basidiomycota
5297	*Bullera alba*	supraglacial sediments	Miage glacier, Mont Blanc, Italian Alps, Italy	25°C	Basidiomycota

5438	*Bullera alba*	snow with superficial sediment	Helbronner peak, Mont Blanc, Italian Alps, Italy	25°C	Basidiomycota
10090	*Cutaneotrichosporon moniliiforme*	Soil close to juniper tree, depth: 10 cm.	Constantine, Algeria	20°C	Basidiomycota
4724	*Cystobasidium laryngis*	subglacial sediments	Sforzellina glacier, Ortles Cevedale group, Italian Alps, Sondrio, Italy	25°C	Basidiomycota
4765	*Cystobasidium laryngis*	glacial ice	Sforzellina glacier, Ortles Cevedale group, Italian Alps, Sondrio, Italy	25°C	Basidiomycota
4772; 4773	*Cystobasidium laryngis*	glacial ice	Forni glacier, Ortles Cevedale group, Italian Alps, Sondrio, Italy	25°C	Basidiomycota
5035	*Cystobasidium laryngis*	superficial glacial sediment	Calderone glacier, Gran Sasso, Abruzzo, Italy	25°C	Basidiomycota
5084; 5088; 5098; 5151	*Cystobasidium laryngis*	deep piping glacial sediments	Calderone glacier, Gran Sasso, Abruzzo, Italy	25°C	Basidiomycota
5287	*Cystobasidium laryngis*	morainic soil	Miage glacier, Mont Blanc, Italian Alps, Italy	25°C	Basidiomycota
5292; 5363; 5366	*Cystobasidium laryngis*	supraglacial sediments	Miage glacier, Mont Blanc, Italian Alps, Italy	25°C	Basidiomycota
3234	*Cystobasidium minutum*		Calabria, Italy	25°C	Basidiomycota

4374	*Cystobasidium minutum*	sea water	Italy	25°C	Basidiomycota
4375	*Cystobasidium minutum*	frozen fish	Italy	25°C	Basidiomycota
4562	*Cystobasidium minutum*	flower of Acanthaceae	Angra dos Reis, Brazil	25°C	Basidiomycota
5227	*Cystobasidium minutum*	soil near the glacier	E.Point, South Valley, Antarctica	25°C	Basidiomycota
5230 - 5232; 5234	*Cystobasidium minutum*	ornithogenic soil	E.Point, South Valley, Antarctica	25°C	Basidiomycota
4845	*Cystofilobasidium capitatum*	glacial melting water	Calderone glacier, Gran Sasso, Abruzzo, Italy	20°C	Basidiomycota
4985; 4987; 4991; 4997; 5002 - 5004; 5008	*Cystofilobasidium capitatum*	superficial glacial melting water	Calderone glacier, Gran Sasso, Abruzzo, Italy	20°C	Basidiomycota
5214	*Cystofilobasidium capitatum*	glacial melting water	Calderone glacier, Gran Sasso, Abruzzo, Italy	20°C	Basidiomycota
4296	*Cystofilobasidium infirmominiatum*	winery surface	Bettona, Perugia, Italy	20°C	Basidiomycota
4884	*Cystofilobasidium infirmominiatum*	surface of *Tuber mescutericum*	Italy	20°C	Basidiomycota
5097	*Filobasidium oeirense*	deep piping glacial sediment	Calderone glacier, Gran Sasso, Abruzzo, Italy	20°C	Basidiomycota
5115	*Filobasidium oeirense*	superficial glacial melting water	Calderone glacier, Gran Sasso, Abruzzo, Italy	20°C	Basidiomycota

4988	*Filobasidium stepposum*	superficial glacial melting water	Calderone glacier, Gran Sasso, Abruzzo, Italy	20°C	Basidiomycota
5222; 5223	*Filobasidium stepposum*	permafrost	Antarctic Dry Valleys, close to the Easter side of the Upper Victoria Lake, Antarctica	20°C	Basidiomycota
5432; 5463	*Filobasidium stepposum*	snow with superficial sediment	Helbronner peak, Mont Blanc, Italian Alps, Italy	20°C	Basidiomycota
5085; 5089; 5095; 5099; 5153 - 5156	*Filobasidium wieringae*	deep piping glacial sediments	Calderone glacier, Gran Sasso, Abruzzo, Italy	20°C	Basidiomycota
5455; 5457	*Filobasidium wieringae*	snow with superficial sediment	Helbronner peak, Mont Blanc, Italian Alps, Italy	20°C	Basidiomycota
4841	*Glaciozyma martinii*	supraglacial sediments	Calderone glacier, Gran Sasso, Abruzzo, Italy	15°C	Basidiomycota
8018	*Glaciozyma martinii*	soil	Sothern Victoria land, Antarctica	15°C	Basidiomycota
4726	*Glaciozyma watsonii*	subglacial sediments	Forni glacier, Ortles Cevedale group, Italian Alps, Sondrio, Italy	15°C	Basidiomycota
4799; 4802	*Glaciozyma watsonii*	subglacial sediments	Sforzellina glacier, Ortles Cevedale group, Italian Alps, Sondrio, Italy	15°C	Basidiomycota

8014; 8015	*Glaciozyma watsonii*	soil	Sothern Victoria land, Antarctica	15°C	Basidiomycota
5194; 5196 - 5199; 5201; 5203	*Goffeauzyma gastrica*	supraglacial sediments	Calderone glacier, Gran Sasso, Abruzzo, Italy	20°C	Basidiomycota
5204 - 5206; 5208 - 5211	*Goffeauzyma gastrica*	ice	Calderone glacier, Gran Sasso, Abruzzo, Italy	20°C	Basidiomycota
5220	*Goffeauzyma gastrica*	glacial melting water	Calderone glacier, Gran Sasso, Abruzzo, Italy	20°C	Basidiomycota
5293 ; 5294	*Goffeauzyma gastrica*	supraglacial sediments	Miage glacier, Mont Blanc, Italian Alps, Italy	20°C	Basidiomycota
5324; 5401	*Goffeauzyma gastrica*	morainic soil	Miage glacier, Mont Blanc, Italian Alps, Italy	20°C	Basidiomycota
5371; 5381	*Goffeauzyma gastrica*	supraglacial sediments	Miage glacier, Mont Blanc, Italian Alps, Italy	20°C	Basidiomycota
4708 - 4710; 4712; 4714; 4733 - 4735; 4737; 4738; 4740 - 4749	*Goffeauzyma gilvescens*	subglacial sediments	Sforzellina glacier, Ortles Cevedale group, Italian Alps, Sondrio, Italy	20°C	Basidiomycota
4750	*Goffeauzyma gilvescens*	endoglacial sediments	Forni glacier, Ortles Cevedale group, Italian Alps, Sondrio, Italy	20°C	Basidiomycota
5924; 10045	*Goffeauzyma gilvescens*	supraglacial sediments	Miage glacier, Mont Blanc, Italian Alps, Italy	20°C	Basidiomycota

5965	*Goffeauzyma gilvescens*	glacial melting water (stagnant)	Miage glacier, Mont Blanc, Italian Alps, Italy	20°C	Basidiomycota
10073	*Goffeauzyma gilvescens*	sediment	Miage glacier, Mont Blanc, Italian Alps, Italy	20°C	Basidiomycota
4837	*Holtermanniella wattica*	supraglacial sediments	Calderone glacier, Gran Sasso, Abruzzo, Italy	20°C	Basidiomycota
5079	*Holtermanniella wattica*	deep piping glacial sediments	Calderone glacier, Gran Sasso, Abruzzo, Italy	20°C	Basidiomycota
5282; 5353; 5408	*Holtermanniella wattica*	morainic soil	Miage glacier, Mont Blanc, Italian Alps, Italy	20°C	Basidiomycota
5411; 5421	*Holtermanniella wattica*	ice cores	Miage glacier, Mont Blanc, Italian Alps, Italy	20°C	Basidiomycota
5998	*Holtermanniella wattica*	supraglacial sediments	Miage glacier, Mont Blanc, Italian Alps, Italy	20°C	Basidiomycota
4794	*Leucosporidium creatinivorum*	supraglacial sediments	Forni glacier, Ortles Cevedale group, Italian Alps, Sondrio, Italy	20°C	Basidiomycota
5217	*Leucosporidium intermedium*	glacial melting water	Calderone glacier, Gran Sasso, Abruzzo, Italy	20°C	Basidiomycota
4867	*Leucosporidium scottii*	surface of Tuber indicatum	China	20°C	Basidiomycota

4979; 4990; 4994; 4999; 5000	*Mrakia aquatica*	superficial glacial melting water	Calderone glacier, Gran Sasso, Abruzzo, Italy	20°C	Basidiomycota
5179; 5180	*Mrakia cryoconiti*	supraglacial sediments	Calderone glacier, Gran Sasso, Abruzzo, Italy	20°C	Basidiomycota
5302	*Mrakia cryoconiti*	superficial glacial melting water	Miage glacier, Mont Blanc, Italian Alps, Italy	20°C	Basidiomycota
4977; 4983; 4995	*Mrakia gelida*	superficial glacial melting water	Calderone glacier, Gran Sasso, Abruzzo, Italy	15°C	Basidiomycota
5106	*Mrakia gelida*	deep piping glacial sediments	Calderone glacier, Gran Sasso, Abruzzo, Italy	15°C	Basidiomycota
5306; 5308, 5952; 5953	*Mrakia gelida*	glacial melting water	Miage glacier, Mont Blanc, Italian Alps, Italy	15°C	Basidiomycota
4728; 4762; 4790	*Mrakia robertii*	subglacial sediments	Forni glacier, Ortles Cevedale group, Italian Alps, Italy	15°C	Basidiomycota
5309	*Mrakia robertii*	glacial melting water	Miage glacier, Mont Blanc, Italian Alps, Italy	15°C	Basidiomycota
5336; 5345	*Mrakia robertii*	morainic soil	Miage glacier, Mont Blanc, Italian Alps, Italy	15°C	Basidiomycota
5922; 5923; 5926	*Mrakia robertii*	supraglacial sediments	Miage glacier, Mont Blanc, Italian Alps, Italy	15°C	Basidiomycota

4819; 5187; 5193; 5195	*Naganishia adeliensis*	supraglacial sediments	Calderone glacier, Gran Sasso, Abruzzo, Italy	20°C	Basidiomycota
5081; 5149; 5152	*Naganishia adeliensis*	deep piping glacial sediments	Calderone glacier, Gran Sasso, Abruzzo, Italy	20°C	Basidiomycota
5275; 5299; 5300; 5321; 5338; 5394	*Naganishia adeliensis*	morainic soil	Miage glacier, Mont Blanc, Italian Alps, Italy	20°C	Basidiomycota
5296; 5382	*Naganishia adeliensis*	supraglacial sediments	Miage glacier, Mont Blanc, Italian Alps, Italy	20°C	Basidiomycota
5967; 5973; 10001	*Naganishia adeliensis*	supraglacial melting water	Miage glacier, Mont Blanc, Italian Alps, Italy	20°C	Basidiomycota
10060	*Naganishia adeliensis*	sediment	Miage glacier, Mont Blanc, Italian Alps, Italy	20°C	Basidiomycota
4910 - 4915	*Naganishia albida*	truffle, Tuber aestivum, associated with Quercus pubescens	Volperino, Perugia, Italy	25°C	Basidiomycota
4917 - 4919	*Naganishia albida*	soil	Volperino, Perugia, Italy	25°C	Basidiomycota
4939; 4940	*Naganishia albida*	mycorrhizae of *Tuber aestivum* associated with *Corylus avellana*	Volperino, Perugia, Italy	25°C	Basidiomycota
4942	*Naganishia albida*	soil around *Corylus avellana* in a truffle field, *Tuber aestivum*	Volperino, Perugia, Italy	25°C	Basidiomycota

3184	*Naganishia albida*	flowers	Venarotta, Ascoli Piceno, Italy	25°C	Basidiomycota
3190	*Naganishia albida*			25°C	Basidiomycota
4345	*Naganishia albida*	sea water	Italy	25°C	Basidiomycota
5270 - 5274; 5276 - 5278; 5281; 5283; 5314 - 5316; 5318; 5322	*Naganishia antarctica*	morainic soil	Miage glacier, Mont Blanc, Italian Alps, Italy	20°C	Basidiomycota
4736	*Naganishia vaughanmartiniae*	supraglacial sediments	Sforzellina glacier, Ortles Cevedale group, Italian Alps, Sondrio, Italy	20°C	Basidiomycota
4584; 4601	*Papiliotrema laurentii*	blackberry, *Rubus* sp.	Horco Molle, Tucuman, Argentina	25°C	Basidiomycota
4586; 4588; 4592; 4596	*Papiliotrema laurentii*	contaminated water near a dam	El Frontal Dam, Sgo del Estero, Argentina	25°C	Basidiomycota
4593; 4594; 4600; 4602; 4603; 4605	*Papiliotrema laurentii*	red fruit	Horco Molle, Tucuman, Argentina	25°C	Basidiomycota
4597; 4598	*Papiliotrema laurentii*	Flores borravino	Horco Molle, Tucuman, Argentina	25°C	Basidiomycota
5449	*Papiliotrema laurentii*	snow with superficial sediment	Helbronner peak, Mont Blanc, Italian Alps, Italy	25°C	Basidiomycota
5006	*Rhodosporidiobolus colostri*	superficial glacial melting water	Calderone glacier, Gran Sasso, Abruzzo, Italy	20°C	Basidiomycota

5982	*Rhodosporidiobolus colostri*	supraglacial melting water	Miage glacier, Mont Blanc, Italian Alps, Italy	20°C	Basidiomycota
3984; 3985	*Rhodotorula glutinis*	caverns		25°C	Basidiomycota
3044; 4304	*Rhodotorula glutinis*			25°C	Basidiomycota
3090	*Rhodotorula glutinis*	grape must	Conegnano, Veneto, Italy	25°C	Basidiomycota
3235; 3236	*Rhodotorula glutinis*	Limburger cheese	Wageningen, The Netherlands	25°C	Basidiomycota
3380	*Rhodotorula glutinis*	flowers	Venarotta, Ascoli Piceno, Italy	25°C	Basidiomycota
3442; 3443	*Rhodotorula glutinis*	milk	Italy	25°C	Basidiomycota
4529; 4611	*Rhodotorula glutinis*	water of *Quesnelia quesneliana*, mangrove	Coroa Grande, Brazil	25°C	Basidiomycota
4949; 4964	*Rhodotorula glutinis*	mature domestic compost	Perugia, Italy	25°C	Basidiomycota
4958	*Rhodotorula glutinis*	commercial compost, 16 m into the process (Temp. 59°C)	Pietramelina, Perugia, Italy	25°C	Basidiomycota
4530	*Rhodotorula glutinis*	flower of Poaceae	Tijuca forest, Brazil	25°C	Basidiomycota
4531	*Rhodotorula glutinis*	flower of *Pachystachys riedeliana*, Acanthaceae	Botanical garden, Rio de Janiero, Brazil	25°C	Basidiomycota

4620	*Rhodotorula graminis*	soil at 850 m alt., 2 cm depth	Bromide Oklahoma, USA	25°C	Basidiomycota
4203	*Rhodotorula mucilaginosa*	vineyard soil	Italy	25°C	Basidiomycota
4376	*Rhodotorula mucilaginosa*	frozen chicken	Italy	25°C	Basidiomycota
4377; 4408	*Rhodotorula mucilaginosa*	sea water	Italy	25°C	Basidiomycota
4378	*Rhodotorula mucilaginosa*	frozen fish	Italy	25°C	Basidiomycota
4618; 4629	*Rhodotorula mucilaginosa*	soil at 850 m alt., 2 cm depth	Bromide Oklahoma, USA	25°C	Basidiomycota
4898	*Rhodotorula mucilaginosa*	surface of *Tuber indicatum*	China	25°C	Basidiomycota
4920; 4945 - 4947	*Rhodotorula mucilaginosa*	*Tuber aestivum* associated with *Corylus avellana*	Volperino, Perugia, Italy	25°C	Basidiomycota
4950; 4952; 4956; 4957	*Rhodotorula mucilaginosa*	mature domestic compost	Perugia, Italy	25°C	Basidiomycota
4962; 4963	*Rhodotorula mucilaginosa*	commercial compost, 15 m into the process (Temp. 63°C)	Pietramelina, Perugia, Italy	25°C	Basidiomycota
5235	*Rhodotorula mucilaginosa*	soil near the glacier	E.Point, North Valley, Antarctica	25°C	Basidiomycota
4466	*Rhodotorula mucilaginosa*	lemon skin	Italy	25°C	Basidiomycota
4536	*Rhodotorula mucilaginosa*	flower of *Ixora coccinea*, Rubiaceae, infested by	Botanical garden, Rio de Janiero, Brazil	25°C	Basidiomycota

		insects			
4669	*Rhodotorula mucilaginosa*	epicarp of *Tuber magnatum* Pico	Campello su Clitunno, Perugia, Italy	25°C	Basidiomycota
3045	*Rhodotorula mucilaginosa*	selected starter for cheese production	Italy	25°C	Basidiomycota
3237	*Rhodotorula mucilaginosa*	grape must	Calabria, Italy	25°C	Basidiomycota
3238; 3239	*Rhodotorula mucilaginosa*	Limburger cheese	Wageningen, The Netherlands	25°C	Basidiomycota
3381; 3382	*Rhodotorula mucilaginosa*	flowers	Venarotta, Ascoli Piceno, Italy	25°C	Basidiomycota
3383; 3384	*Rhodotorula mucilaginosa*	flowers	Perugia, Italy	25°C	Basidiomycota
3444 - 3446	*Rhodotorula mucilaginosa*	milk	Italy	25°C	Basidiomycota
3538	*Rhodotorula mucilaginosa*	soil	Ultuna, Sweden	25°C	Basidiomycota
5839	*Solicoccozyma aeria*	soil close to plum tree, depth: 35 cm. Agricultural soil: presence of important vegetation, some fallen fruit (plum) rotting and insects	District of Hamma Bouziane, Constantine, Algeria	20°C	Basidiomycota
5150	*Solicoccozyma aeria*	deep piping glacial sediments	Calderone glacier, Gran Sasso, Abruzzo, Italy	20°C	Basidiomycota

5291; 5367	*Solicoccozyma aeria*	supraglacial sediments	Miage glacier, Mont Blanc, Italian Alps, Italy	20°C	Basidiomycota
5690	*Solicoccozyma aeria*	snow from trench	Helbronner peak, Mont Blanc, Italian Alps, Italy	20°C	Basidiomycota
10019	*Solicoccozyma aeria*	supraglacial sediments	Miage glacier, Mont Blanc, Italian Alps, Italy	20°C	Basidiomycota
10094	*Solicoccozyma aeria*	soil close to eucalyptus tree, depth: 10 cm. Forest soil: presence of important vegetation, some insects	Constantine, Algeria	20°C	Basidiomycota
5344; 5403	*Solicoccozyma terreus*	morainic soil	Miage glacier, Mont Blanc, Italian Alps, Italy	20°C	Basidiomycota
4812; 4814	*Solicoccozyma terricola*	subglacial sediments	Forni glacier, Ortles Cevedale group, Italian Alps, Sondrio, Italy	20°C	Basidiomycota
5280; 5311 - 5313; 5317; 5319; 5320; 5341; 5346; 5350	*Solicoccozyma terricola*	morainic soil	Miage glacier, Mont Blanc, Italian Alps, Italy	20°C	Basidiomycota
5368; 5870; 5907; 5925; 10017	*Solicoccozyma terricola*	supraglacial sediments	Miage glacier, Mont Blanc, Italian Alps, Italy	20°C	Basidiomycota

5224	*Sporobolomyces metaroseus*	permafrost	Antarctic Dry Valleys, close to the Easter side of the Upper Victoria Lake, Antarctica	20°C	Basidiomycota
4387	*Sporobolomyces roseus*	sea water	Italy	25°C	Basidiomycota
5010; 5018; 5020; 5022; 5029; 5120	*Sporobolomyces roseus*	superficial glacial sediment	Calderone glacier, Gran Sasso, Abruzzo, Italy	25°C	Basidiomycota
3254 - 3258	*Tausonia pullulans*	Limburger cheese	The Netherlands	20°C	Basidiomycota
4822; 4836; 5172	*Tausonia pullulans*	supraglacial sediments	Calderone glacier, Gran Sasso, Abruzzo, Italy	20°C	Basidiomycota
4969; 4970; 4872; 5007	*Tausonia pullulans*	superficial glacial melting water	Calderone glacier, Gran Sasso, Abruzzo, Italy	20°C	Basidiomycota
5105; 5108; 5170; 5171	*Tausonia pullulans*	deep piping glacial sediments	Calderone glacier, Gran Sasso, Abruzzo, Italy	20°C	Basidiomycota
5215	*Tausonia pullulans*	glacial melting water	Calderone glacier, Gran Sasso, Abruzzo, Italy	20°C	Basidiomycota
5298; 5871	*Tausonia pullulans*	supraglacial sediments	Miage glacier, Mont Blanc, Italian Alps, Italy	20°C	Basidiomycota
5873	*Tausonia pullulans*	glacial melting water	Miage glacier, Mont Blanc, Italian Alps, Italy	20°C	Basidiomycota

5959	*Tausonia pullulans*	glacial melting water (stagnant)	Miage glacier, Mont Blanc, Italian Alps, Italy	20°C	Basidiomycota
10065; 10070; 10071	*Ustilentyloma graminis*	sediment	Miage glacier, Mont Blanc, Italian Alps, Italy	20°C	Basidiomycota
4923 - 4838	*Vanrija humicola*	*Tuber aestivum* associated with *Corylus avellana*	Volperino, Perugia, Italy	25°C	Basidiomycota
4941; 4943	*Vanrija humicola*	soil around *Corylus avellana* in a truffle field, *Tuber aestivum*	Volperino, Perugia, Italy	25°C	Basidiomycota
5111	*Vishniacozyma tephrensis*	deep piping glacial sediment	Calderone glacier, Gran Sasso, Abruzzo, Italy	20°C	Basidiomycota
5475	*Vishniacozyma tephrensis*	snow with superficial sediment	Helbronner peak, Mont Blanc, Italian Alps, Italy	20°C	Basidiomycota
4826	*Vishniacozyma victoriae*	*Tuber aestivum* associated with *Corylus avellana*	Volperino, Perugia, Italy	20°C	Basidiomycota
4830; 4835	*Vishniacozyma victoriae*	deep piping glacial sediments	Calderone glacier, Gran Sasso, Abruzzo, Italy	20°C	Basidiomycota
4965; 4967	*Vishniacozyma victoriae*	superficial glacial melting water	Calderone glacier, Gran Sasso, Abruzzo, Italy	20°C	Basidiomycota
4968; 4973; 4978; 4986; 5013; 5015; 5017; 5055 - 5057; 5132	*Vishniacozyma victoriae*	superficial glacial melting water	Calderone glacier, Gran Sasso, Abruzzo, Italy	20°C	Basidiomycota

5207; 5212; 5301	*Vishniacozyma victoriae*	ice	Calderone glacier, Gran Sasso, Abruzzo, Italy	20°C	Basidiomycota
5354	*Vishniacozyma victoriae*	morainic soil	Miage glacier, Mont Blanc, Italian Alps, Italy	20°C	Basidiomycota
