# Supplementary figures and images for: Study of Holtermanniella wattica, Leucosporidium creatinivorum, Naganishia adeliensis, Solicoccozyma aeria, and Solicoccozyma terricola for their lipogenic aptitude from different carbon sources
Source: Biotechnol Biofuels. 2016 Nov 28;9:259. doi: 10.1186/s13068-016-0672-1 (PMC5126845; doi:10.1186/s13068-016-0672-1)

## Additional file 2

Flow chart figure reporting the workflow of the study

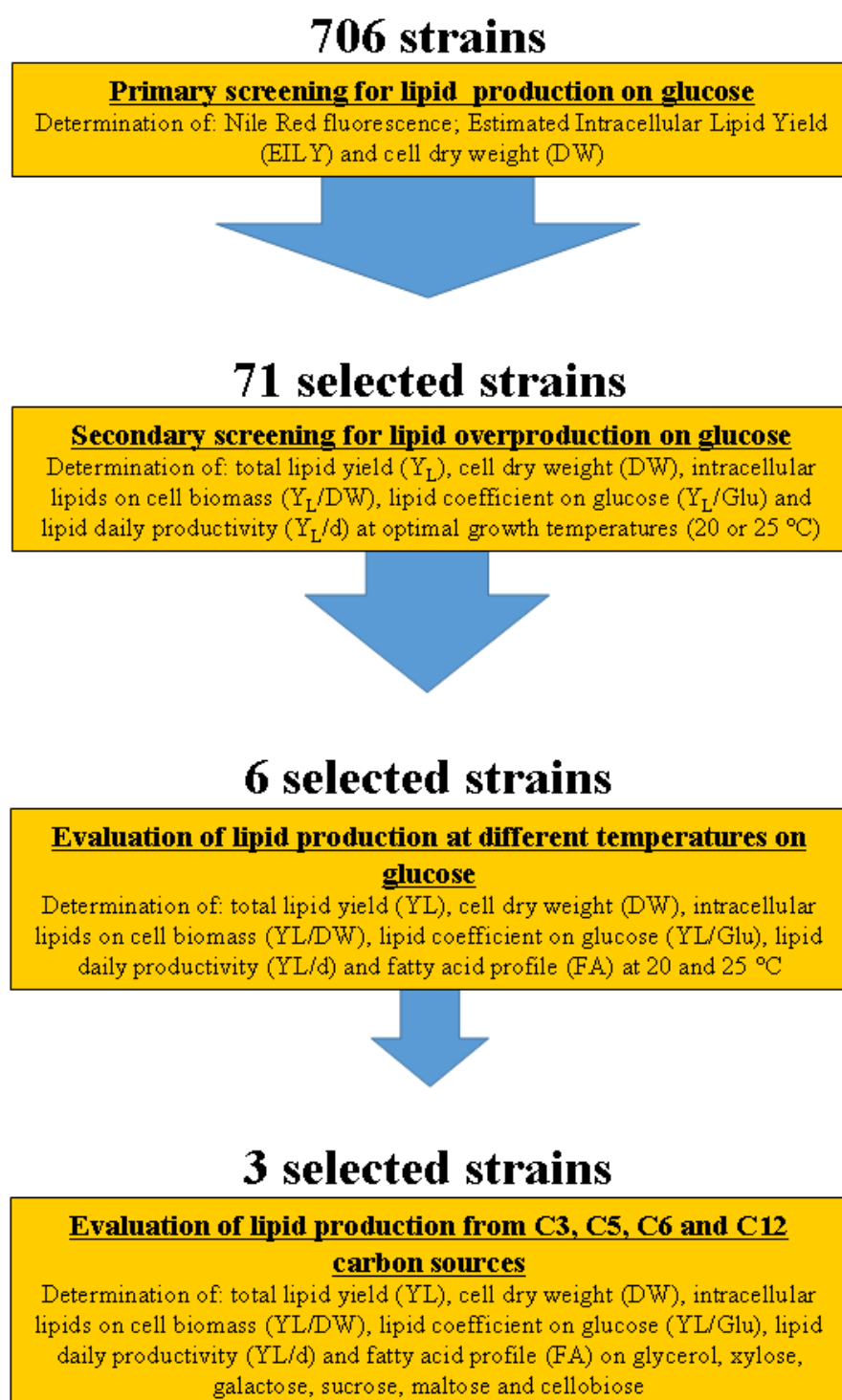

Supplement: Supplementary file 2 — Additional file 2: Figure S1. Flow chart figure reporting the workflow of the study. A flow chart figure summarizing the workflow of the study. [file 13068_2016_672_MOESM2_ESM.pdf]
