# Supplementary material for: Study of Holtermanniella wattica, Leucosporidium creatinivorum, Naganishia adeliensis, Solicoccozyma aeria, and Solicoccozyma terricola for their lipogenic aptitude from different carbon sources
Source: Biotechnol Biofuels. 2016 Nov 28;9:259. doi: 10.1186/s13068-016-0672-1 (PMC5126845; doi:10.1186/s13068-016-0672-1)
Supplement: Supplementary file 3 — Additional file 3: Figure S2. A few examples of micrographs of yeast strains before and during fluorescence emission. Increasing intracellular lipid yield evaluated by Nile Red before (A, C, E and G) and during fluorescence emission (B, D, F and H) photographed with a Wild MP 552 camera (Leica). A and B = Debaryomyces hansenii DBVPG 3326; C and D = Zygosaccharomyces bisporus DBVPG 3018; E and F = Magnusiomyces capitatus DBVPG 3250; G and H = Naganishia albida DBVPG 4919. [file 13068_2016_672_MOESM3_ESM.pdf]

Additional file 3

A few examples of micrographs of yeast strains before and during fluorescence emission.

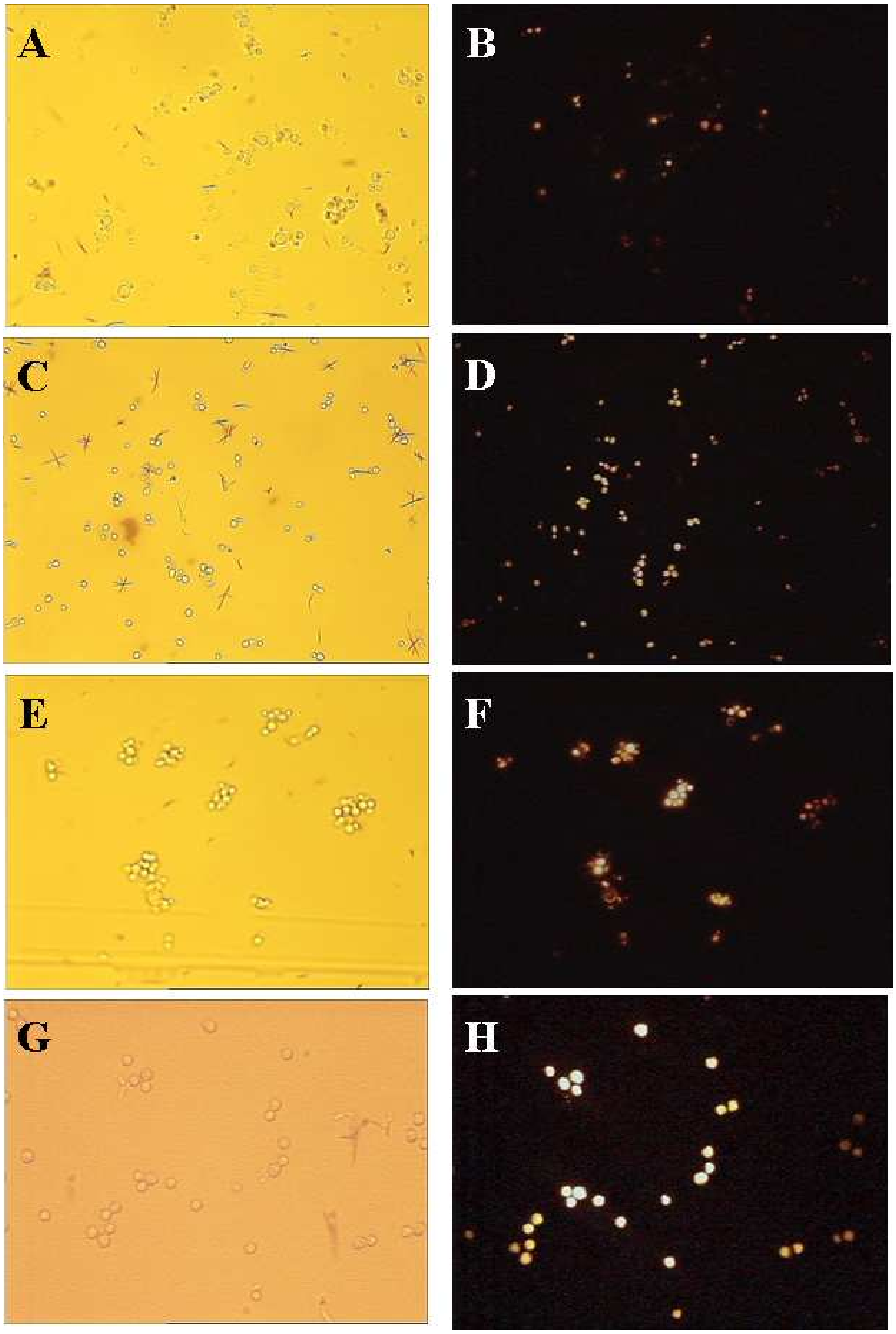

Increasing intracellular lipid yield evaluated by Nile Red before (A, C, E and G) and during fluorescence emission (B, D, F and H) photographed with a Wild MP 552 camera (Leica). A and B = *Debaryomyces hansenii* DBVPG 3326; C and D = *Zygosaccharomyces bisporus* DBVPG 3018; E and F = *Magnusiomyces capitatus* DBVPG 3250; G and H = *Naganishia albida* DBVPG 4919.
