# Supplementary material for: Study of Holtermanniella wattica, Leucosporidium creatinivorum, Naganishia adeliensis, Solicoccozyma aeria, and Solicoccozyma terricola for their lipogenic aptitude from different carbon sources
Source: Biotechnol Biofuels. 2016 Nov 28;9:259. doi: 10.1186/s13068-016-0672-1 (PMC5126845; doi:10.1186/s13068-016-0672-1)
Supplement: Supplementary file 4 — Additional file 4: Figure S3. High resolution images of NR staining of Leucosporidium creatinivorum DBVPG 4794 (incubated at 20 °C), Naganishia adeliensis DBVPG 5195 and Solicoccozyma terricola DBVPG 5870 (both at 25 °C). High resolution images of Nile Red staining of Naganishia adeliensis DBVPG 5195 (A and B, incubated at 25 °C), Solicoccozyma terricola DBVPG 5870 (C and D, 25 °C) and Leucosporidium creatinivorum DBVPG 4794 (E and F, 20 °C). Photographs were captured before (A, C and E) and during fluorescence emission (B, D and F) with an UV epifluorescence microscope Olympus BX53 (Olympus Co., Centre Valley, PA, USA) equipped with excitation filter BP470-500 and barrier filter BA515-560 and a XC50 camera (Olympus Co.). [file 13068_2016_672_MOESM4_ESM.pdf]

Additional file 4

High resolution images of NR staining of *Leucosporidium creatinivorum* DBVPG 4794 (incubated at 20°C), *Naganishia adeliensis* DBVPG 5195 and *Solicoccozyma terricola* DBVPG 5870 (both at 25°C).

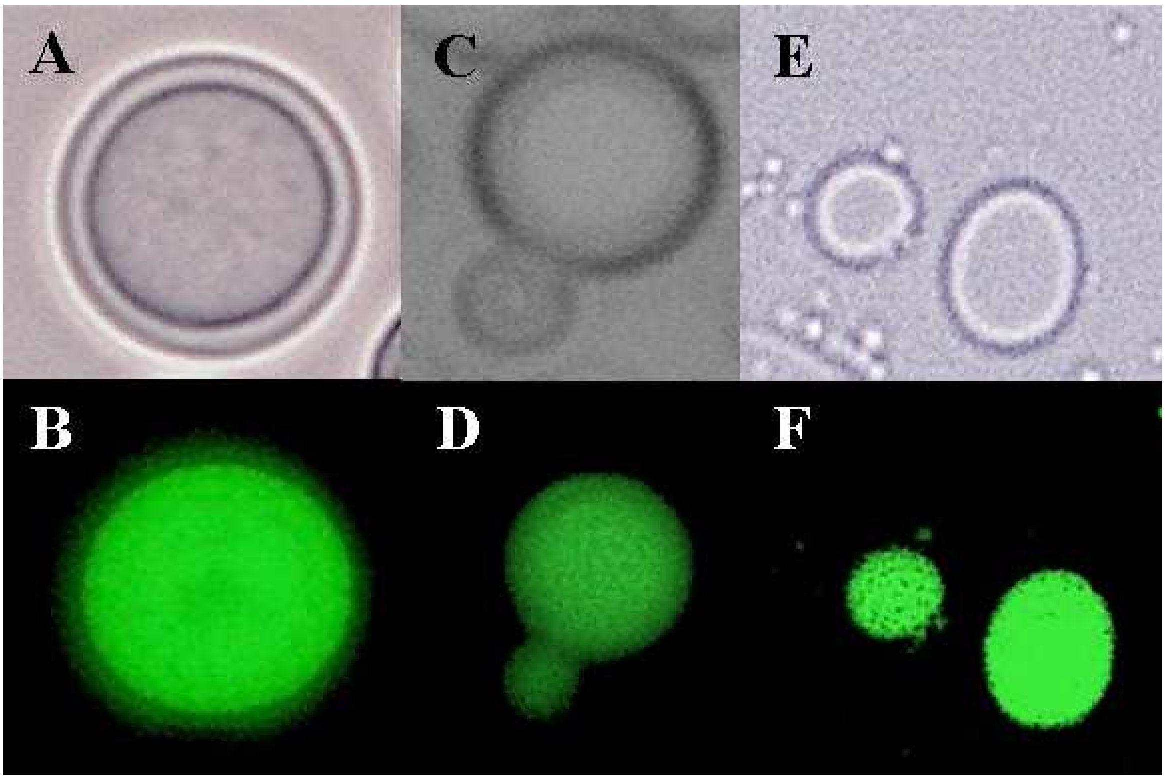

High resolution images of Nile Red staining of *Naganishia adeliensis* DBVPG 5195 (A and B, incubated at 25°C), *Solicoccozyma terricola* DBVPG 5870 (C and D, 25°C) and *Leucosporidium creatinivorum* DBVPG 4794 (E and F, 20°C). Photographs were captured before (A, C and E) and during fluorescence emission (B, D and F) with an UV epifluorescence microscope Olympus BX53 (Olympus Co., Centre Valley, PA, USA) equipped with excitation filter BP470-500 and barrier filter BA515-560 and a XC50 camera (Olympus Co.).
