# Supplementary material for: Study of Holtermanniella wattica, Leucosporidium creatinivorum, Naganishia adeliensis, Solicoccozyma aeria, and Solicoccozyma terricola for their lipogenic aptitude from different carbon sources
Source: Biotechnol Biofuels. 2016 Nov 28;9:259. doi: 10.1186/s13068-016-0672-1 (PMC5126845; doi:10.1186/s13068-016-0672-1)
Supplement: Supplementary file 5 — Additional file 5: Figure S4. 2D scatterplot of EILY vs DW of 706 yeast strains studied. Estimated Intracellular Lipid Yield (EILY, calculated as reported in the text) and cell dry weight (DW) of the strains herein studied assessed by a Kruskal–Wallis test (P < 0.05). A comparison of EILY and DW in dependence of taxonomic position (Ascomycota vs Basidiomycota) of the 706 strains. Red symbols = ascomycetous strains; black symbols = basidiomycetous strains. A and B = centroids of ascomycetous and basidiomycetous strains, respectively. B comparison of EILY and DW in dependence of optimal growth temperature (psychrophiles vs psychrotolerant vs mesophiles) of 422 basidiomycetous strains. Red symbols = psychrophilic strains; blue symbols = psychrotolerant strains; black symbols = mesophilic strains. P, Pt and M = centroids of psychrophilic, psychrotolerant and mesophilic strains, respectively. [file 13068_2016_672_MOESM5_ESM.pdf]

**Additional File 5**

2D scatterplot of EILY *vs* DW of 706 yeast strains studied.

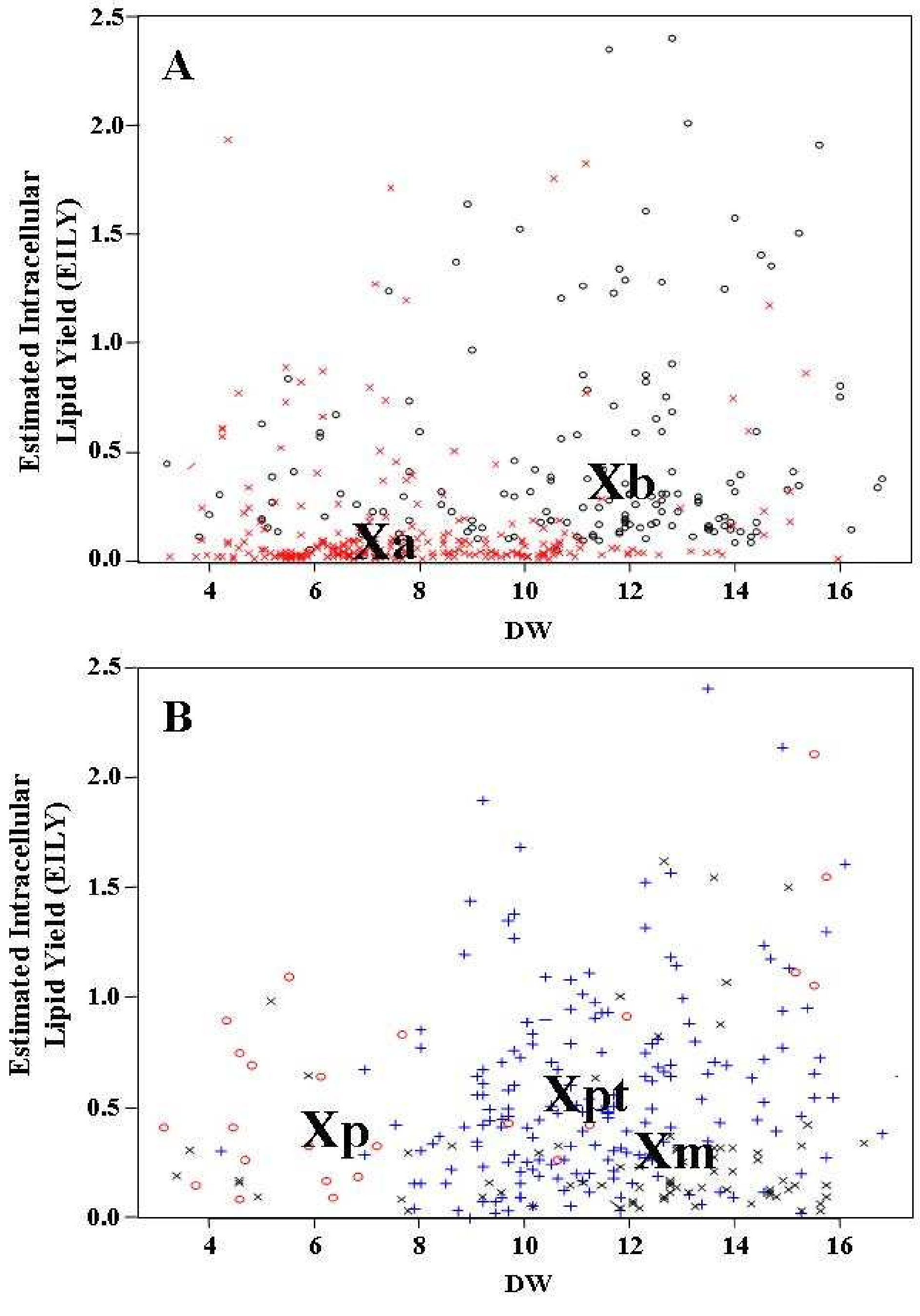


Estimated Intracellular Lipid Yield (EILY, calculated as reported in the text) and cell dry weight (DW) of the strains herein studied assessed by a Kruskal-Wallis test ($P < 0.05$). (A): comparison of EILY and DW in dependence of taxonomic position (Ascomycota vs Basidiomycota) of the 706 strains. Red symbols = ascomycetous strains; black symbols = basidiomycetous strains. A and B = centroids of ascomycetous and basidiomycetous strains, respectively. (B): comparison of EILY and DW in dependence of optimal growth temperature (psychrophiles vs psychrotolerant vs mesophiles) of 422 basidiomycetous strains. Red symbols = psychrophilic strains; blue symbols = psychrotolerant strains; black symbols = mesophilic strains. P, Pt and M = centroids of psychrophilic, psychrotolerant and mesophilic strains, respectively.
