# Supplementary material for: Study of Holtermanniella wattica, Leucosporidium creatinivorum, Naganishia adeliensis, Solicoccozyma aeria, and Solicoccozyma terricola for their lipogenic aptitude from different carbon sources
Source: Biotechnol Biofuels. 2016 Nov 28;9:259. doi: 10.1186/s13068-016-0672-1 (PMC5126845; doi:10.1186/s13068-016-0672-1)
Supplement: Supplementary file 6 — Additional file 6: Figure S5. Time course of lipid production of Leucosporidium creatinivorum DBVPG 4794 (grown at 20 °C), Naganishia adeliensis DBVPG 5195 and Solicoccozyma terricola DBVPG 5870 (both at 25 °C). YL = Total lipid yield; DW = cell dry weight; YL/DW = total intracellular lipids on cell biomass; YL/Glu = lipid coefficient. [file 13068_2016_672_MOESM6_ESM.pdf]

Additional file 6

Time course of lipid production of *Leucosporidium creatinivorum* DBVPG 4794 (grown at 20°C), *Naganishia adeliensis* DBVPG 5195 and *Solicoccozyma terricola* DBVPG 5870 (both at 25°C).

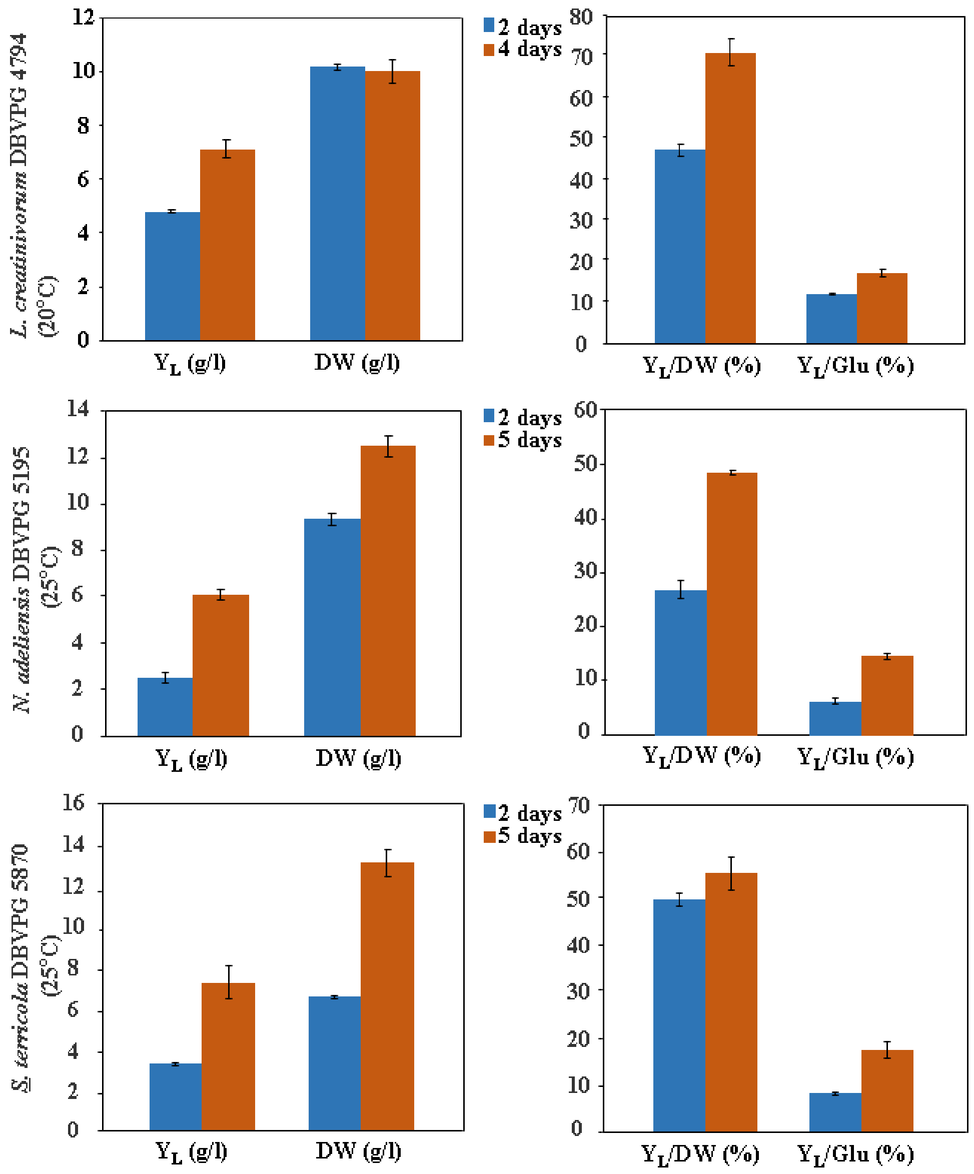

Y_L = Total lipid yield; DW = cell dry weight; Y_L/DW = total intracellular lipids on cell biomass; Y_L/Glu = lipid coefficient.
